# Robustness of connectome harmonics to local gray matter and long-range white matter connectivity changes

**DOI:** 10.1016/j.neuroimage.2020.117364

**Published:** 2021-01-01

**Authors:** Sébastien Naze, Timothée Proix, Selen Atasoy, James R. Kozloski

**Affiliations:** a IBM T.J. Watson Research Center, Yorktown Heights, New York, USA; b IBM Research Australia, Melbourne, Victoria, Australia; c Department of Basic Neurosciences, Faculty of Medicine, University of Geneva, Geneva, Switzerland; d Department of Psychiatry, University of Oxford, UK

## Abstract

Recently, it has been proposed that the harmonic patterns emerging from the brain's structural connectivity underlie the resting state networks of the human brain. These harmonic patterns, termed *connectome harmonics*, are estimated as the Laplace eigenfunctions of the combined gray and white matters connectivity matrices and yield a connectome-specific extension of the well-known Fourier basis. However, it remains unclear how topological properties of the combined connectomes constrain the precise shape of the connectome harmonics and their relationships to the resting state networks. Here, we systematically study how alterations of the local and long-range connectivity matrices affect the spatial patterns of connectome harmonics. Specifically, the proportion of local gray matter homogeneous connectivity versus long-range white-matter heterogeneous connectivity is varied by means of weight-based matrix thresholding, distance-based matrix trimming, and several types of matrix randomizations. We demonstrate that the proportion of local gray matter connections plays a crucial role for the emergence of wide-spread, functionally meaningful, and originally published connectome harmonic patterns. This finding is robust for several different cortical surface templates, mesh resolutions, or widths of the local diffusion kernel. Finally, using the connectome harmonic framework, we also provide a proof-of-concept for how targeted structural changes such as the atrophy of inter-hemispheric callosal fibers and gray matter alterations may predict functional deficits associated with neurodegenerative conditions.

## 1 Introduction

Understanding the structure-function relationships in large-scale brain networks is an active research topic in neuroscience (Sporns, Chialvo, Kaiser, Hilgetag, 2004, Honey, Thivierge, Sporns, 2010, Mii, Betzel, de Reus, van den Heuvel, Berman, McIntosh, Sporns, 2016). In clinical neuroscience, these relationships can elucidate the role of structural changes in neurological diseases and their respective symptoms. Graph theoretical analysis of brain connectivity has led to new insights about cortical wiring patterns (Jirsa, McIntosh, 2007, Bullmore, Sporns, 2009) such as small-world topology, presence of hubs, hierarchical properties, and enabled the development of quantitative measures of network resilience (Rubinov and Sporns, 2010). These metrics are now commonly used to understand the organization of brain function and dysfunction (Fornito et al., 2015), including Alzheimer’s disease (Stam, De Haan, Daffertshofer, Jones, Manshanden, van Cappellen van Walsum, Montez, Verbunt, De Munck, Van Dijk, 2008, Stam, Jones, Nolte, Breakspear, Scheltens, 2006), dementia (Agosta et al., 2013; Vecchio et al., 2015), schizophrenia (Alexander-Bloch et al., 2013; Gollo, Roberts, Cropley, Di Biase, Pantelis, Zalesky, Breakspear, 2018, van den Heuvel, Mandl, Stam, Kahn, Pol, 2010, van den Heuvel, Sporns, Collin, Scheewe, Mandl, Cahn, Goi, Pol, Kahn, 2013, Lynall, Bassett, Kerwin, McKenna, Kitzbichler, Muller, Bullmore, 2010) and Huntington’s disease (Harrington et al., 2015; McColgan et al., 2015).

Several studies have applied the graph Laplacian, a discrete version of the continuous Laplace operator, to brain connectivity matrices and inferred progression of neurodegenerative diseases (Raj et al., 2012), brain malformation (Wang et al., 2017), time of attention switching in a cognitive task (Huang et al., 2018; Medaglia et al., 2018), macroscale coupling gradient between brain regions (Preti and Van De Ville, 2019), and altered dynamic connectivity in patients with concussion (Sihag et al.. 2020). These studies focus on long range white-matter connectivity and use brain connectivity matrices derived from parcellation schemes that range from a few dozens to several hundred regions-of-interests (ROI) (Desikan et al., 2006; Destrieux et al., 2010). Recently, another framework has been proposed for the application of graph Laplacian to the human connectome, wherein the local connectivity of the gray matter cortical structure estimated from the magnetic resonance imaging (MRI) data is combined with the long-range connectivity of the white-matter thalamo-cortical fibers estimated from the diffusion MRI (dMRI) data without incorporating any parcellation of the cortical surface (Atasoy, Donnelly, Pearson, 2016, Atasoy, Deco, Kringelbach, Pearson, 2017). This densely sampled connectome model has the advantage of offering the minimum amount of discretization possible in the given resolution of the MRI and dMRI data and yielding the closest approximation of the continuous Laplace operator, as the graph Laplacian converges to its continuous counterpart when the number of uniformly sampled data points taken from the underlying manifold increases (Belkin and Niyogi, 2008). In contrast to other parcellation-based approaches, the input structure of this framework consists of a graph, where each node represents a vertex from the cortical surface mesh without applying any parcellation. Hence, this approach provides an increase in the number of nodes by two orders of magnitude compared to other methods utilising brain parcellations. The eigenvectors of this dense connectome Laplacian (called ”connectome harmonics”) yield a set of frequency-ordered harmonic patterns emerging on the cortex and provide a connectome-specific extension of the well-known Fourier basis to the human brain. Connectome harmonics produced by this framework suggest a relationship between low frequency harmonics and brain's resting state networks such as the default mode network (DMN) and reveal unique and frequency-specific changes in brain activity measured by functional magnetic resonance imaging (fMRI) data (Atasoy, Roseman, Kaelen, Kringelbach, Deco, Carhart-Harris, 2017, Atasoy, Vohryzek, Deco, Carhart-Harris, Kringelbach, 2018).

However, connectome reconstruction methods generally suffer from many caveats that hinder the accurate and reproducible reconstruction of connectomes (Jbabdi and Johansen-Berg, 2011; Jones et al., 2013). Here, we aim to provide a systematic assessment of connectome harmonic patterns by quantifying their sensitivity to variations of structural connectivity, and apply the resulting framework to characterize brain changes during neurodegeneration. We demonstrate the effects of different framework parameters on the emergence of reliable connectome harmonics using widely acknowledged and validated template datasets. By systematically altering properties of the local connectivity of various cortical surfaces, characteristics of long-range connections, and the proportion of local and long-range connectivities, we evaluate the sensitivity of the framework to produce reliable connectome harmonics. Our results reveal that both local gray matter connectivity and long-distance white matter fiber tracts determine the exact shape of the connectome harmonic patterns, yet the local gray matter connectivity plays a crucial role in the emergence of functionally meaningful spatial patterns on the cortical surface. Our sensitivity analysis further extends previous applications of connectome harmonics by introducing structural white matter and gray matter alterations to observe their consequences on functional patterns emerging on the cortical surface. Finally, we also discuss implications of the presented connectome alterations possibly relating for existing disease conditions such as Huntington’s and other neurodegenerative disorders.

## 2 Methods

### 2.1 Multi-modal imaging framework and dataset

**Fig. 1:**
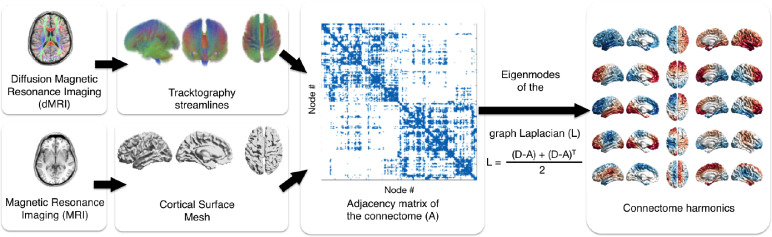
**Overview of the workflow for the construction of the connectome harmonics.** Local connectivity from cortical surface mesh (bottom left) and long-range connections from tractography (top left) are combined in a high-resolution structural connectome (middle), from which a graph Laplacian *L* is computed based on the adjacency (*A*) and the degree (*D*) matrices of the combined connectivities. Connectome harmonics (right) are the eigenvectors of the graph Laplacian.

We implemented the systematic construction of connectome harmonics (Fig. 1), that we integrated into an existing processing pipeline (SCRIPTS, (Proix et al., 2016)). Briefly, SCRIPTS is an open-source pipeline that processes MRI and dMRI to build subject-specific surface meshes, parcellations and corresponding connectivity matrices. We extended this pipeline with new features allowing for computing subject-specific and subject-averaged high-resolution surface-based connectivity matrices, and connectome harmonics. The framework combines local and long-range connectivity matrices to form a high-resolution structural connectome, on which the graph Laplacian is applied, and from which the connectome harmonics are computed. Only parameters relevant to the high-resolution connectome and the construction of those harmonics are explored in this work.

To alleviate the known shortcomings associated with subject-specific imaging methods (Bürgel et al., 2006; Willats et al., 2014) from our study, we used validated template datasets from open-source studies to generate our results. For the surface mesh, we used subject-averaged FreeSurfer templates *cvs_avg35_in_MNI152* (default), of 20,000 vertices, as well as *fsaverage5* (20,484 vertices) and *fsaverage4* (5,124 vertices). All surfaces were registered in the MNI space so the impact of different mesh resolutions could be assessed without manual intervention. For the white-matter streamlines, we used the Gibbs dataset, which contains 20,712,081 streamlines computed by probabilistic tractography from 169 subjects (Horn and Blankenburg, 2016). All streamlines are registered in MNI coordinates and normalized to minimize inter-subject differences of brain sizes and shapes. The dataset has been cross validated across cortico-cortical and cortico-thalamo-cortical atlases (Behrens et al., 2003; Bürgel et al., 2006; Horn and Blankenburg, 2016). See **Supplementary Text 1** for further details about the acquisition and processing of images from the Gibbs connectome database, whereby individual subjects were registered in MNI space and normalized using the DARTEL procedure (Ashburner, 2007) in order to correct for inter-subject differences in brain volume and estimate a group connectome.

### 2.2 Local and long-range connectivity

Gray matter intracortical connectivity includes both branched axonal ramifications within the gray matter (typically at lengths of 0−2 mm (Braitenberg and Schuz, 2013), and the intrinsic horizontal connectivity that can reach up to 8 mm within the gray matter along unbranched axons that run parallel to the cortical surface (Gonzlez-Burgos, Barrionuevo, Lewis, 2000, Melchitzky, GonzlezBurgos, Barrionuevo, Lewis, 2001, Voges, Guijarro, Aertsen, Rotter, 2010, Voges, Schüz, Aertsen, Rotter, 2010, Braitenberg and Schuz, 2013). The horizontal gray matter connections can terminate in patches across brain regions, resulting in anisotropic propagation of brain activity throughout the gray matter and thus playing a role in first order processing among sensory areas (Voges and Perrinet, 2012). White matter connectivity is usually derived from dMRI and tractography analysis and includes both streamlines of lengths less than 40 mm (Bajada et al., 2019), including U fibers, known to be unreliably counted due to limitations in dMRI spatial resolution (Gigandet et al., 2008), and medium and long-range streamlines that are 80−160 mm long, believed to be associated with areas involved in functional integration (Bajada et al., 2019). Note that to show U-fibers as short as 3 mm requires a specific tractography algorithm parameter of step size less than 1 mm and a high resolution MRI, which differs from the standard protocol (Song et al., 2014). In our study, we use the term *local connectivity* to refer to intrinsic horizontal gray matter connectivity of length 1−6 mm, but not including the single cortical column scale of less that 1 mm. This choice is consistent with the cortical surfaces used herein, whereby the average edge lengths are 2.9 mm, 3.1 mm and 5.6 mm for *fsaverage5, cvs_avg35* and *fsaverage4*, respectively (see also **Suppl. Figure 1C**). We use the term *long-range connectivity* to refer to any white matter connection fibers with a minimum track length of 10 mm. This lower bound of 10 mm for a tractography step size of 2 mm is consistent with previous seminal work wherein tracks shorter than 5 mm were discarded when a tractography step size of 1 mm was used (Zalesky et al., 2010) in order to avoid the inclusion of spurious tracks (see **Suppl. Figure 1B**). The shortest of the white matter connections are referred to as short-range white matter connections, but should not be confused with the local gray matter connectivity described above.

### 2.3 Computation of high-resolution connectome

We computed the vertex-based high-resolution connectome using streamlines reconstructed by tractography and vertices from the cortical surface meshes. We manually checked that tracks and mesh were properly aligned (see **Suppl. Figure 1A**), for distance between track bounds and nearest cortical surface mesh (see **Suppl. Figure 1B**). Intersections between the streamlines with FreeSurfer’s cortical surface mesh templates were assessed using the coordinates of two points of the tracks at each bound (see **Suppl. Figure 2**). If an intersection was found for each bounds *i* and *j*, the connection weight *C*_*i,j*_ between the vertices nearest to the intersection points is incremented by 1. Otherwise, if for one or both bounds the track was too short to reach the surface mesh (no intersection point), a linear extension of 3 mm using the direction defined by linearly interpolating the bound and the third last coordinates from the bound was added to the track, and the intersection was assessed again. If no intersection is found in one or both bounds even after extension, the track is discarded. Length of track bound and size of the extension are adjustable parameters, and several cortical meshes are available using white-matter gray-matter boundary (WMGM) or gray matter (pial) surfaces.

### 2.4 Computation of connectome harmonics

As in (Atasoy et al., 2016), we use the graph Laplacian *L* as the discrete counterpart of the Laplace operator applied to the brain connectivity matrix. The graph Laplacian can be interpreted as a particular case of the discrete approximation of the continuous Laplace-Beltrami operator, a generalization of the Laplace operator to Riemannian manifolds. We use the graph Laplacian (Levy, 2006) defined as:

(1) $$L=\frac{1}{2}\left(\left(D-A\right)+{\left(D-A\right)}^{T}\right),$$

where *A* is the adjacency matrix of the *combined* connectome consisting of white-matter and gray-matter connectivity, and *D* its degree matrix. Note that other formulae exist for computing the discrete Laplacian. Our choice of this Laplacian formula is mainly driven by its numerical stability as discussed in (Levy, 2006). The *combined* connectome is the adjacency matrix *A* obtained by combining the local connectivity adjacency matrix *A*_ℓ_ derived from the cortical surface mesh and the long-range connectivity adjacency matrix *A_c_* constructed by tractography methods:

(2) $$A=A_{\ell }\cup A_{c},$$

whereby both *A*_ℓ_ and *A_c_* are *m* × *m* matrices with *m* being the number of vertices of the cortical surface mesh. The long-range connectivity adjacency matrix *A_c_* is derived by removing the weakest weights of the z-scored long-range connectivity matrix *C^z^* (referred in this article as thresholding):

(3) $$\begin{array}{c} A_{c_{i,j}}=\left\{ \begin{array}{cc} 1 & \text{if}\;C_{i,j}^{z}>z_{{}_{C}}\;,\\ 0 & \text{otherwise}\; \end{array}\right. \end{array}$$

for all i,j=1,…,m, with $z_{C}$ being the adjacency weight threshold. Note that we applied z-score normalization to the long-range connectivity matrix *C* in order to have integer values of $z_{{}_{C}}$ relating to the standard deviation of connection weights:

(4) $$C^{z}=\left(C-\mu_{{}_{C}}\right)/\sigma_{{}_{C}},$$

where $\mu_{{}_{C}}$ is the mean connectivity strength of the long-range connectome *C*, and $\sigma_{{}_{C}}$ its standard deviation. *A*_ℓ_ represents the local gray matter connectivity matrix. As in (Atasoy et al., 2016), two nodes are locally connected when they are connected through the mesh as direct neighbors.

We then decompose the graph Laplacian *L* into a finite number of eigenvalues *λ_k_* and eigenvectors, or connectome harmonics, *ψ_k_*:

$$L\;\psi_{k}=\lambda_{k}\psi_{k}.$$

### 2.5 Connectome alterations

Local and long-range connectivities can be altered by changing parameters at several stages of the framework and are summarized in Table 1. At early stages, smoothing of the cortical surface mesh (*f_i_*) and the number of streamlines to retain from tractography are important parameters to consider in building connectivity matrices (we used by default all available streamlines). At later stages, different parameter selections are possible for creating adjacency matrices from weighted connectivity matrices by thresholding (*z_C_*), for trimming (*η, κ*) of the long-range white matter connections, and for the diffusion kernel width (Λ_*s*_), mesh resolution (*n_v_*), and anisotropy (*ρ*) of the local gray matter connections. These parameters influence the outcome of the framework by modifying the graph structure, the proportion of local or long-range connections in the combined connectome used for computing harmonics, or selectively removing specific connections or connectivity patterns.

**Table 1:** Summary table of parameters.

Parameter	Default value; [range]	Unit	Interpretation
*f_i_*	0; [0; 8; 21; 89]	⌀	Smoothing coefficient of the cortical surface mesh
*z_C_*	1; [0–10]	standard deviation of weight distribution	Adjacency weight threshold used for binarization of long-range connectivity matrix
Λ_*s*_	2; [1; 2]	node distance (number of graph edges away)	Width of the local connectivity kernel
*η*	0; [0–100]	%	Percentage of long-range connection trimmed based on averaged white matter track length
*κ*	0 [0–100]	%	Percentage of interhemispheric long-range connections randomly removed (*callosectomy*)
*ρ*	0 [0–100]	%	Percentage of local connections randomly removed (*anisotropy*)

Default parameter values were chosen based on a preliminary parameter space exploration so that harmonics could be generated most reliably while varying a subset of other parameters. Mapping of the Default Mode Network (DMN) to the Desikan-Killiany atlas is provided in **Suppl. Table 1**.

#### 2.5.1 Local connectivity alterations

*Cortical surface mesh smoothing.* Cortical surface meshes are often averaged or smoothed in studies looking at averaged brain properties (Fischl et al., 1999). Here, smoothing was performed to soften the strong curvature of the surface prior to computing the connectome by the surface-tracts intersection routine (see **Suppl. Figure 2**). Because linearly extended tracts can terminate obliquely or not cross the mesh, this routine can result in a reassignment of the track bound to a different cortical surface node, or the track not being assigned at all, for computing the brain wide connectivity matrix. Here when indicated, cortical surface meshes from Freesurfer’s templates were smoothed using the smoothPatch function from an open source MATLAB toolbox (https://www.mathworks.com/matlabcentral/fileexchange/26710-smooth-triangulated-mesh) using the Laplacian smoothing with inverse vertice-distance based weights and by varying the smoothing coefficient *f_i_*. The numbers of smoothing iterations were taken from the Fibonacci sequence, up to 89 iterations, and visually inspected.

*Local diffusion kernel width and mesh resolution.*We applied two widths Λ_*s*_ of diffusion kernels over the cortical surface to compute the local gray matter connectivity matrix (Atasoy et al., 2016). Widths Λ_*s*_ comprised either one or two nearest neighbors, each resulting in a different proportion of local to long-range connection when the number of long-range white matter connections are fixed. If instead the proportion of local to long-range connections was held constant, a larger local diffusion kernel width allows to increase the number of long-range connections incorporated to the combined connectome. For example, a local:long-range proportion of 1: 1 with a local kernel of only immediate neighbors resulted in a combined connectome comprising around 100,000 long-range connections. The same 1: 1 proportion with a local kernel spanning immediate neighbors and their neighbors resulted in a combined connectome comprising around  400,000 long-range connections. The default local kernel width chosen throughout the study was two, but the results do not change with only direct neighbor connections (Fig. 8). To vary the mesh resolution, we used a mesh with 20,484 vertices (*fsaverage5*) and a coarser version of 5,124 vertices (*fsaverage4*). Changes in mesh resolution affect the position of vertices and can result in a different vertex attribution of track bounds or in discarded tracks.

*Anisotropy.*We introduce the removal of cortical surface mesh edges in a process termed *anisotropy*, whereby mesh edges are removed either randomly (with probability *ρ*), by ascending order, or by descending order of edge lengths. A visualization of the resulting graphs structure for gradual changes to *ρ* is provided in **Suppl. Fig. 10** for illustrative purpose.

#### 2.5.2 Long-range connectivity alterations

We performed two distinct operations to alter the long-range connectivity: thresholding, which is based on the number of tracks between vertices (weight-based), and trimming, which is based on the average track length between vertices (distance-based).

*Long-range connectivity thresholding.*For thresholding, the z-scored weighted long range connectivity matrix *C^z^* (Eq. 4) was binarized according to an adjacency weight threshold value $z_{C}$. The ratio *r* of local versus long-range connections is defined as $r=\text{tr}\left(A_{\ell }\right)/\;\left(\text{tr}\left(A_{\ell }\right)+\text{tr}\left(A_{c}\right)\right)$, which is the number of local connections divided by the summed numbers of local and long-range connections. Because the local gray matter connectivity matrix is determined by the cortical surface mesh and the diffusion kernel, we kept it constant (unless otherwise mentioned) and varied the white matter connectivity threshold $z_{{}_{C}},$ which thereby controls the proportion *r* of local connections. As weights represent the number of streamlines connecting distant nodes, when $z_{C}$ increases, only the most prominent tracks of the white matter remain.

*Long-range connectivity trimming.*Trimming was simulated by removing some percentage *η* of the long range connectivity entries based on their average track length. Different scenarios were implemented: *1)* removing the longest tracks first; *2)* removing the shortest tracks first; and *3)* removing tracks in random order. Note that we applied trimming to the long-range white matter connections after applying the threshold $z_{C}=1,$ thereby setting the proportion of local connections to *r* ≃ 0.7. Thus, the reported percentage of trimming affected only the remaining long-range white matter connections after thresholding.

*Callosectomy.*Finally, we introduce the removal of inter-hemispheric connections in a process termed *callosectomy*, whereby inter-hemispheric connections are removed either randomly (with probability *κ*) or by descending order of track lengths. A visualization of the resulting graphs structure for gradual values of *κ* is provided in **Suppl. Figure 10** for illustrative purpose.

### 2.6 Comparison metrics

To assess the sensitivity of a given parameter of the framework, we compared connectome harmonics by computing Pearson correlations between the harmonic patterns emerging from unaltered and altered connectomes. We also compared each harmonic to the DMN using mutual information as in (Atasoy et al., 2016). These metrics serve different purposes: Pearson correlation permits quantification of similarity between harmonics, while mutual information indirectly measures the structure-function relationships between each harmonic and the DMN.

#### 2.6.1 Correlation

Each connectome harmonic is represented by a vector of the size of the number of vertices in a cortical surface mesh. When smoothing the cortical surface mesh or changing its resolution, the ordering of vertices is affected. To overcome such disorganization, the harmonic vectors are projected onto the coarser space of the Desikan-Killiany atlas, which is conserved by region mapping. We refer to this coarser atlas space as the *lower resolution* alternative to the *higher resolution* mesh space. The similarity between harmonics of two different meshes is then assessed in the *lower resolution* atlas space by computing the correlation *P* between the two coarse harmonic vectors. As two sets of connectome harmonics become identical, a very high correlation value results (*P* → 1) for pairs of harmonics with the same index, results in a diagonal harmonics correlation matrix (Fig. 6, 7 and 8). Divergence from the diagonal matrix reflects a decrease in correlation between harmonics of the same index across the two sets, reflecting a disorganization of the eigen-decomposition output in the form of eigenvalue/eigenvector pairs.

#### 2.6.2 Mutual information

The Mutual Information (MI) between a connectome harmonic *ψ_k_* and the DMN vector *v*_DMN_ was computed as follows:

(5) $$MI\left(\psi_{k},v_{\mathrm{DMN}}\right)=\sum_{n=1}^{N}\sum_{m=1}^{M}p_{\psi_{k},v_{\mathrm{DMN}}}\left(n,m\right)\log(\frac{p_{\psi_{k},v_{\mathrm{DMN}}}\left(n,m\right)}{p_{\psi_{k}}\left(n\right)p_{\mathrm{DMN}}\left(m\right)})$$

where *p*(*n*) is the probability distribution of *n*, and *p*(*n, m*) is the joint probability distribution of *n* and *m*, with *v*_DMN_ being the vector representing the Default Mode Network in atlas space. The values of the harmonic vector were discretized into N=16 bins, while the DMN is represented by a binary vector (M=2 bins), whether the cortical mesh vertex is part of the DMN or not (Atasoy et al., 2016). Mapping of the Default Mode Network (DMN) to the Desikan-Killiany atlas is provided in **Suppl. Table 1**.

### 2.7 Surrogate and statistics

Different surrogates of the connectome harmonics were obtained by using different methodological types of randomization of the high-resolution connectome. We used the Monte-Carlo method with the null hypothesis that harmonics estimated from the structural connectivity matrix after randomizing the long-range connectivity (randomized harmonics ${\tilde{\psi }}_{k}$) have the same similarity, as measured by MI, compared to the ones generated from the original connectome. Each type of randomization was repeated 100 times, involved shuffling long-range but not local connectivity, and was performed using the brain connectivity toolbox (Rubinov and Sporns, 2010) wherein a selected ratio of connections are shuffled while keeping the degree of each node unchanged (i.e. the total number of connections per node remained the same for each node). This constraint maintains the hubness of the graph, and as such is not as destructive to graph features as is a completely unconstrained shuffling method. Allowing for hubness deterioration was not within the current scope of work but may be investigated in the future. We term the randomization type involving shuffling only inter-hemispheric connections of the long-range connectivity matrix *inter*, and randomization involving shuffling only intra-hemispheric connections of the long-range connectivity matrix *intra*. For *inter+intra* randomization, both inter- and intra-hemispheric connections were shuffled by isolating each quadrant of the matrix and shuffling them independently. For *global* randomization, all long-range connections are shuffled in a single batch.

We computed a surrogate summary statistic by calculating the probability, $p_{{}_{MI_{\text{surr}}}}$, that the $MI_{\text{surr}}$ being greater than the original $MI_{\text{orig}}$ for $N_{\text{surr}}$ surrogates of a subset of *n_k_* consecutive connectome harmonics $\psi_{k\in K=\{k_{0},\cdots ,k_{0}+n_{k}\}}$, starting at rank *k*_0_. In the most common case through this manuscript, $k_{0}=7$ and $n_{k}=5$ as we are interested in harmonics 7 to 11.

$$p_{{}_{{MI}_{\text{surr}}}}=\frac{1+\sum_{n=1}^{N_{\text{surr}}}\sum_{m=0}^{n_{k}-1}\left\{ \begin{array}{cc} 1 & \text{if}\;MI_{{\text{orig}}_{k_{0}+m}}\leq MI_{{\text{surr}}_{n,k_{0}+m}}\\ 0 & \text{otherwise} \end{array}\right.}{1+N_{\text{surr}}\times n_{k}}$$

where $N_{\text{surr}}=100$ is the number of surrogates for each randomization type. Statistics were computed for all values of $z_{C}$ but only reported for $z_{C}=1$ since it is the critical value for which large scale patterns emerge on the cortical surface. We corrected p-values for multiple comparisons using the Benjamini-Hochberg procedure with false-discovery rate 0.1 (McDonald, 2009).

## 3 Results

To understand the effect of the two different types of structural connectivities composing the human connectome as defined in (Atasoy et al., 2016), namely the local gray matter connectivity and the long-range white matter connectivity, we altered several parts of the framework influencing either the long-range white connectivity only, local gray matter connectivity only, or both together.

### 3.1 Effects of white matter connection changes on connectome harmonics, affecting only long-range connectivity

#### ***Weight-based thresholding***

**Fig. 2:**
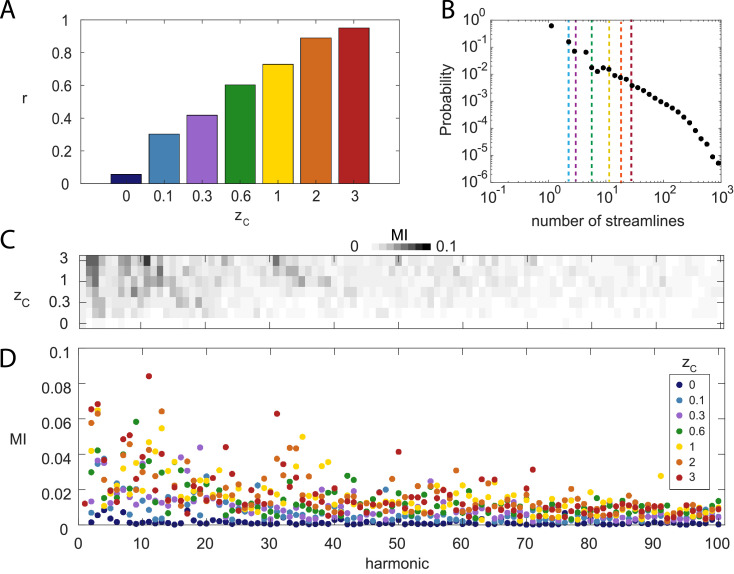
**Weight-based thresholding of long-range white matter connections increases MI between connectome harmonics and the default mode network (DMN)**. (A) Proportion *r* of local gray matter connections in the adjacency matrix *A* of the graph Laplacian, for different threshold value *z_C_* applied to the long-range white matter connectivity. (B) Distribution of connectome weights (number of white matter streamlines *W* between vertices) in log-log scale, described as probability given that the weight is positive (*P*(*W*|*W* > 0)). Vertical lines correspond to $z_{C}$ values with same color code as A). (C-D) Mutual information (MI) between the first 100 connectome harmonics and the DMN for a range of *z_C_* resulting in a range of proportion *r* of local connections. Color code is consistent across panels. (See **Suppl. Figure 3** for MI with other RSNs).

We first investigated the effect of applying different threshold values *z_C_* to the weights of the long-range white-matter connections included in the human connectome (see *Methods*). As expected, the ratio $r=\text{tr}\left({A_{\ell }}^{2}\right)/(\text{tr}\left({A_{\ell }}^{2}\right)+\text{tr}\left({A_{c}}^{2}\right))$ of the number of local over total number of connections increases as a function of the adjacency weight thresholds $z_{C}$ (Fig. 2A) applied to the white matter connectivity. This leads to the incremental removal of long-range connections with lowest weights (Fig. 2B). We computed the mutual information (MI) between each connectome harmonic pattern and the DMN, projected onto the cortical surface mesh (see *Methods*). We observed that for low frequency connectome harmonics, the MI generally increases with increasing $z_{C}$ (Fig. 2C). This suggests that local connections play a key role in the emergence of functionally relevant connectome harmonic patterns. For high proportions of local over long-range connectivity (e.g., resulting from *z_C_* > 0.6), connectome harmonics $\psi_{k\in K=\{2,3,4,7,8,9,10,11\}}$ yielded the highest MI indicating the strongest overlap of these harmonics with the DMN. These MI values (MI  ≃  0.05) are consistent with the ones previously reported in (Atasoy et al., 2016). To prove the robustness of the framework, we also reproduced those findings in a single HCP subject (see **Suppl. Figure 5A-D**).

Our work focuses on the DMN, but the MI measured between harmonics and other resting state networks (RSN) follows a similar pattern (**Suppl. Figure 3**), whereby higher *z_C_* leads to higher MI. Interestingly, a first peak is commonly observed across several RSNs around the 3^rd^ harmonic, and a broader second peak is observed before or around the 9^th^, which seems more specific to each RSN. A third peak between the 30^th^ and 40^th^ harmonics is present for the DMN. In the following, our network of interest remains the DMN, and the MI is systematically reported for harmonics $\psi_{k\in K=\{7,8,9,10,11\}}$ as motivated by the range reported to match the DMN pattern in (Atasoy et al., 2016) and Fig. 2.

#### ***Randomizations***

We then assessed whether the specific organization of the long-range connections significantly affects the connectome harmonic patterns and their relation to the DMN.

**Fig. 3:**
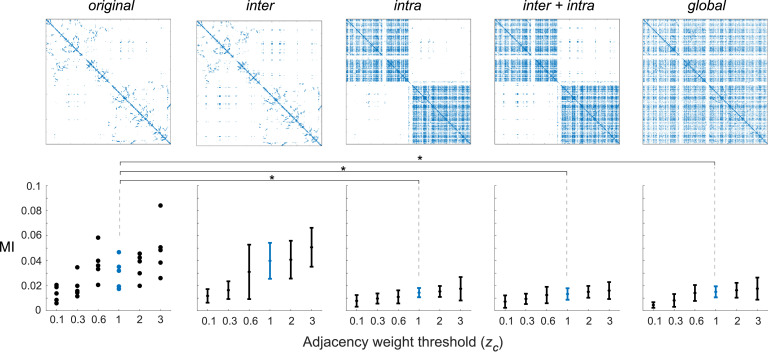
**Mutual Information (MI) between connectome harmonics and the DMN for several randomized versions of long-range white matter connectivity.** Mean and standard deviation of connectome harmonics’ $\psi_{k\in K=\{7,8,9,10,11\}}$ MIs for different proportions of local to long-range connections (parameterized by the adjacency weight threshold $z_{C}$ as in Figure 2). Different types of connectome surrogates are shown: *original* (no randomization), *inter* (interhemispheric only), *intra* (intrahemispheric only), *inter+intra* (inter and intrahemispheric separately), and *global* (inter and intrahemisphere combined), see *Methods*). ⋆ indicates p<0.05 of Monte-Carlo statistical test of $\psi_{k\in K=\{7,8,9,10,11\}}$ MI values with the DMN for original harmonics vs. surrogate data MI distributions for $z_{C}=1$ of the different types of randomizations. MI with other RSNs for those randomizations are shown in **Suppl. Figure 9**.

We generated surrogate data with several types of randomizations of the long-range connections (i.e. *inter, intra, inter+intra*, and *global*, see *Methods*) for each value of $z_{C}$ in order to study their effects on MI between connectome harmonics and the DMN. Local connectivity was kept fixed and MIs between randomized connectome harmonics ${\tilde{\psi }}_{k\in K=\{7,8,9,10,11\}}$ and the DMN were computed (Fig. 3). As previously, long-range connections were eliminated by increasing the threshold *z_C_* (and therefore the ratio *r*) which is entirely dependent on the number of streamlines (weight) of each long-range white matter connection, with weaker connections removed first.

The difference of MI between the original connectome harmonics $\psi_{k\in K=\{7,8,9,10,11\}}$ and the DMN compare to those same harmonics when inter-hemispheric connections are randomized, without significant difference ($\forall z_{C},p>0.1;$ Fig. 3 *original* vs *inter*). However, as soon as intra-hemispheric connections are randomized, the MI with the DMN decreases significantly (p<0.05 for *original* vs. *intra, original* vs. *inter+intra*, and *original* vs. *global*, FDR corrected, reported for $z_{C}=1$ for clarity, corresponding to a proportion *r* ≃ 0.7 of local connections). **Suppl. Figure 7** shows the resulting first 10 harmonics projected onto the cortical surface mesh for visualization of the obtained spatial patterns for those different types of randomizations.

Regardless of the randomization method, MI increases with higher proportions of local to long-range connections (as in Fig. 2). **Suppl. Figure 8** shows that these results are also observed when the MI is computed on the 3^rd^ harmonic alone or a wider range of harmonics (e.g. from 2 to 20, ${\tilde{\psi }}_{k\in K=\{2\ldots 20\}}$). Together, these observations suggest that functionally relevant features of the low-frequency connectome harmonic patterns observed for proportions of local connections *r* > 0.4 (corresponding to *z_C_* > 0.3) disappear with intrahemispheric and global randomization. This is further supported by **Suppl. Figure 9**, showing that these observations are valid not only for the DMN but also for other RSNs. We mainly report the relation to the DMN for consistency with the original study (Atasoy et al., 2016).

#### ***Distance-base trimming***

We investigated the role of shorter versus longer streamlines in the white matter connectivity by removing the long-range connections based on their streamlines average lengths, a process that we call trimming.

**Fig. 4:**
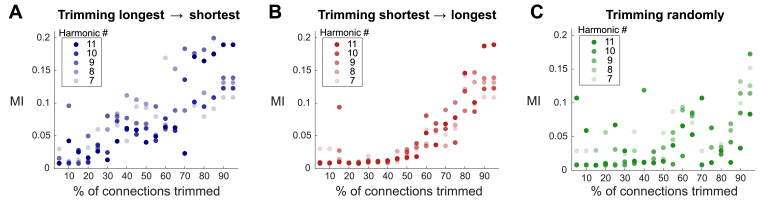
**Effect of different white matter fiber lengths on the emergence of functional harmonics patterns.** Mutual information (MI) between the DMN and connectome harmonics’ $\psi_{k\in K=\{7,8,9,10,11\}}$ for different percentages of trimming of the white matter connectivity. Trimming was performed by eliminating longest tracks first **(A)**, shortest tracks first **(B)**, and in random order **(C)**. Baseline connectome used for $z_{C}=1$ corresponding to *r* ≃ 0.7 before trimming.

When the longest white-matter fibers were trimmed first (Fig. 4A), the MI between connectome harmonics $\psi_{k\in K=\{7,8,9,10,11\}}$ and the DMN showed a rapid increase (i.e. for 0 –40% white matter connections cut), whereas when shortest connections were trimmed first (Fig. 4B) we observed low MI values until a certain threshold was crossed corresponding to  ~ 60% of white matter connections being removed. This result indicates that the presence of long-range streamlines is a main contributing factor for the observed low MI between harmonics and the DMN. Trimming long range connections in random order also showed lower MI values for 0–40% cut (Fig. 4C), and further increases of MI values above 0.05 for trimming above 60%. This is not surprising, since the distribution of fiber lengths is skewed towards the short fibers (see **Suppl. Figure 1B**, middle histogram), leading to a higher probability of removing shorter streamlines first, and thus confirming our findings reported above.

#### ***Callosectomy***

**Fig. 5:**
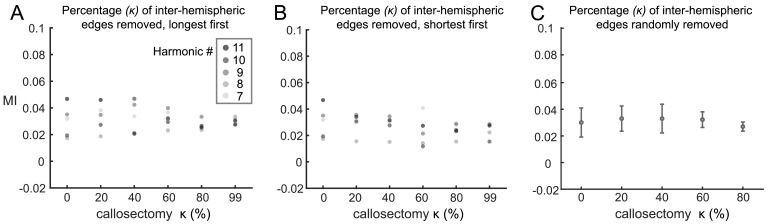
**Mutual Information between connectome harmonics and the DMN for gradual removal of inter-hemispheric white matter connectivity**. Mutual information (MI) between DMN and harmonics $\psi_{k\in K=\{7,8,9,10,11\}}$ for gradual trimming of inter-hemispheric connections (termed *callosectomy*) by descending (A), ascending (B) orders of track length, and randomly (C). Adjacency weight threshold is set to $z_{C}=1$, corresponding to the proportion *r* ≃ 0.7 of local connections before alteration. ⋆ indicates p<0.05 of Monte-Carlo statistical test between distributions of MIs using 100 samples. Note that we used a maximum callosectomy of 99% in order to avoid totally disconnected hemispheres.

Next, we assessed the contribution of inter-hemispheric connections to the functional relation of connectome harmonics to the DMN (Fig. 5). Inter-hemispheric connections were trimmed by ascending and descending fiber lengths or randomly in a process referred to as *callosectomy* (as in Wang et al., 2017). Note that the alteration based on length is deterministic, while the random removal is not, and thus can be run multiple times to establish statistics. Fig. 5 shows that harmonics $\psi_{k\in K=\{7,8,9,10,11\}}$ are not strongly affected by the callosectomy until at least 50% of the inter-hemispheric connections are removed. The relation of harmonics $\psi_{k\in K=\{7,8,9,10,11\}}$ to the DMN is more sensitive to short than long inter-hemispheric fibers when those corresponding connections are remove first (Fig. 5A-B). When randomly trimmed, MI between the DMN and harmonics $\psi_{k\in K=\{7,8,9,10,11\}}$ is not significantly different (p>0.1 using 100 samples, after Bonferroni correction for multiple comparisons). **Suppl. Figure 11** shows the resulting 10 first harmonics projected onto the cortical surface mesh for visualization of the obtained spatial patterns for gradual removal of inter-hemispheric connections.

A special case occurs when all inter-hemispheric connections are removed (i.e. at 100% callosectomy) such that left and right hemispheres are separated and patterns appear consecutively on one hemisphere and the other (**Suppl. Figure 12A**, middle). In such a case, eigenvalues are organized by pairs i.e. $\left(\lambda_{1},\lambda_{2}\right),\left(\lambda_{3},\lambda_{4}\right),\left(\lambda_{5},\lambda_{6}\right),\ldots$ as shown in **Suppl. Figure 12B**, with two zero-valued eigenvalues *λ*_1_ and *λ*_2_ indicating the two isolated sub-graphs (Chung, 1996), one for each hemisphere. As such, we also re-constructed the harmonics on the whole brain by combining eigenvectors computed on each hemisphere separately (**Suppl. Figure 12A**, right). We assessed the MI between these newly generated harmonics and the DMN for different values of white matter connectivity threshold *z_C_* (**Suppl. Figure 12C**) and observe the same pattern of higher MI as *z_C_* increases.

### 3.2 Effects of cortical surface mesh changes on connectome harmonics, affecting both local gray matter and long-range white matter connections

We assessed how changes in the cortical surface mesh geometry and resolution affect the patterns of connectome harmonics.

#### ***Smoothing***

**Fig. 6:**
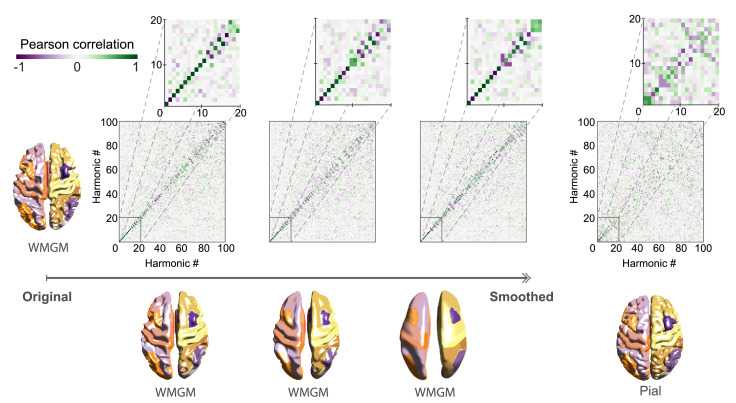
**Low frequency harmonics are robust to cortical surface changes.** High-resolution connectome and connectome harmonics are recomputed for different smoothing levels of WMGM cortical surface (left), or pial surface (right) and compared to original WMGM surface mesh using Pearson correlation in atlas space for the first 100 harmonics. Adjacency weight threshold is set to $z_{C}=1$, corresponding to a proportion *r* ≃ 0.7 of local connections.

Firstly, we investigated the changes in harmonic patterns when using a gray matter surface cortical mesh (Freesurfer’s *pial* surface) versus a white-matter gray-matter boundary surface (WMGM, Freesurfer’s *white* surface (Fig. 6 and **Suppl. Figure 2**). In order to investigate the effect of the precise geometry of the cortex on the particular shape of the connectome harmonic patterns, we applied mesh smoothing to different meshes of the cortical surface and iteratively increased the amount of smoothing. We measured the correlation between harmonics in the atlas space to compare harmonic patterns emerging on different cortical surface meshes (see Methods). Note that smoothing of the cortical surface mesh also affects the white-matter connectome, as the long-range connectivity matrix is generated based on the track-mesh intersections.

We found that when using the WMGM surface mesh, smoothing did not have a significant influence on the lowest frequency harmonics up to the 15^th^, indicated by high diagonal values in the correlation matrices in Fig. 6, constructed by correlating the original cortical surface (rows) and the smoothed surfaces (columns). Higher frequency harmonics were moderately affected by these changes, as seen by high values of correlation shifting from being exactly on the diagonal to being a few elements off diagonal, indicating a re-ordering of eigenvalue-eigenvector pairs by only a few ranks. When using the gray matter pial surface, we observed a degradation of the correlation between harmonic patterns (lower values in the diagonal of the correlation matrix, Fig. 6, right). In this case, correlation values for low frequency harmonics $\psi_{k\in K=\{1,2,3,4,5\}}$ remained unaffected, but decreased for low frequency harmonics $\psi_{k\in K=\{6,\ldots ,20\}}$. Here, low frequency harmonics corresponding to the lowest 20 eigenvalues of the eigenspectrum represent only 0.1% of the harmonic spectrum. Changes were even more prominent in higher frequency modes where the diagonal line disappear. We also reproduced those findings in a single HCP subject (**Suppl. Figure 5E**), showing that the on-diagonal correlation values are very strong including for high-frequency harmonics, which supports that the framework is robust to perturbations of the WMGM surface such as smoothing.

#### ***Mesh resolution***

**Fig. 7:**
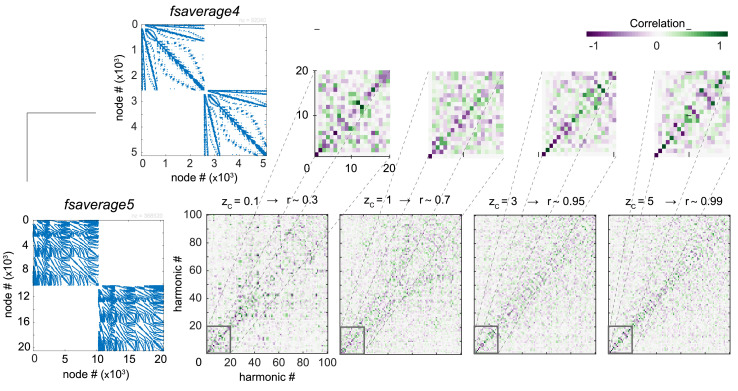
**Low frequency harmonics are robust to mesh resolution changes.** Region-wise correlation of connectome harmonics $\psi_{k\in K=\{1,\ldots ,100\}}$ using *fsaverage4* (5,124 vertices) and *fsaverage5* (20,484 vertices) cortical surface mesh templates from FreeSurfer. Proportion *r* of local connections is indicated by different degrees of connectome adjacency weight threshold $z_{C}$. Insets show a magnified version of the correlation matrix for connectome harmonics $\psi_{k\in K=\{1,\ldots ,20\}}$.

We also examined the impact of using different mesh sizes to represent the cortical surface (Fig. 7), while varying *z_C_* and therefore the proportion of local connections *r*.

We found that when the proportion of local connectivity is high compared to the long-range connections (i.e. *r* > 0.7), the patterns of the emerging low frequency harmonics *ψ*_*k* < 15_ were robust to these changes in local gray matter connectivity, as seen by the diagonal on the correlation matrices for *z_C_* > 1. Note that since different mesh resolution implies that the ordering of vertices from one mesh resolution to the other does not pair, the correlation matrices are computed in atlas space rather than in vertex space, resulting in more noisy values.

### 3.3 Effects of gray matter connection changes on connectome harmonics, affecting only local connectivity

#### ***Local diffusion kernel width***

**Fig. 8:**
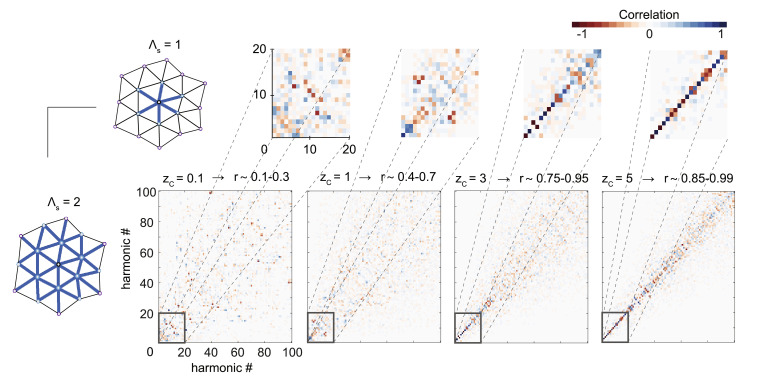
**Low frequency harmonics are robust to local diffusion kernel width changes.** Vertex-wise correlation of connectome harmonics $\psi_{k\in K=\{1,\ldots ,100\}}$ using cvs_avg35 template decimated to 20,000 vertices, using $\Lambda_{s}=1$ (Λ_1_) vs. $\Lambda_{s}=2$ (Λ_2_) neighboring vertices as local connectivity kernel. Proportion *r* of local connections for each kernel sizes (separated by a dash $\Lambda_{1}\;-\;\Lambda_{2}$) is indicated by different degrees of connectome adjacency weight threshold $z_{{}_{C}}$. Insets show a magnified version of the correlation matrix for connectome harmonics $\psi_{k\in K=\{1,\ldots ,20\}}$.

We then focused on alterations affecting only the local gray matter connectivity, first by changing the spatial extent Λ_*s*_ of the local connectivity (Fig. 8) while varying *z_C_* and therefore the proportion of local to long-range connections *r*. As previously reported, we found that when the local connectivity was sufficiently strong with respect to the long-range connections (i.e. *r* > 0.7), the patterns of the emerging low frequency harmonics *ψ*_*k* < 15_ were robust to the widening or narrowing of the local gray matter connectivity kernel width, as seen by a clear diagonal on the correlation matrices for *z_C_* > 1.

Note that the proportion *r* of local gray matter connections also vary according to the local connectivity kernel width Λ_*s*_ since wider kernels create more local connections. We also reproduced those findings in a single HCP subject (**Suppl. Figure 5F**), showing that the on-diagonal correlation values are very strong including for high-frequency harmonics, which supports that the framework is robust to such changes.

#### ***Anisotropy***

**Fig. 9:**
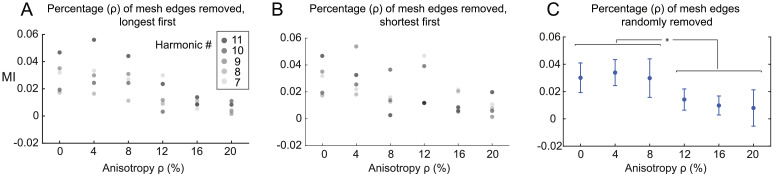
**Mutual Information between connectome harmonics and the DMN for gradual disruptions of local gray matter connectivity**. Mutual information (MI) between DMN and harmonics $\psi_{k\in K=\{7,8,9,10,11\}}$ for gradual removal of local gray matter connections (termed *anisotropy*) by descending (A), ascending (B) order of edge length and in random order (C). Adjacency weight threshold is set to $z_{C}=1$ corresponding to a proportion *r* ≃ 0.7 of local connections before alteration. ⋆ indicates p<0.05 of Monte-Carlo statistical test between distributions of MIs using 100 samples.

The last modification of the local gray matter connectivity consisted of removing edges from the mesh forming the local connectivity matrix, a process termed *anisotropy*. As for the callosectomy, we performed such trimming either by ascending or by descending order of edge lengths, or randomly (Fig. 9). Note again that the alteration based on edge length is deterministic, while the random removal is not and thus can be run multiple times to establish statistics. It can be observed that when edges are removed randomly or when longest edges are removed first (Fig. 9A and 9C), the MI between harmonics $\psi_{k\in K=\{7,8,9,10,11\}}$ and the DMN decreases for anisotropy *ρ* above 10% (p<0.05 using 100 samples, after Bonferroni correction). In the other case, when shortest edges were removed first (Fig. 9B), the MI between harmonics $\psi_{k\in K=\{7,8,9,10,11\}}$ and the DMN decreases faster, for *ρ* of 5%. **Suppl. Figure 7** shows the resulting 10 first harmonics projected onto the cortical surface mesh for visualization of the obtained spatial patterns.

## 4 Discussion

In this work, we have presented a quantitative evaluation of how changes in the structural connectome affect the emergence of connectome harmonic patterns. On one hand, we demonstrate that the proportion of local gray matter to long-range white matter connections is a critical determinant for the emergence of spatial harmonic patterns on the cortical surface consistent with functional brain networks. On the other hand, changes to the cortical surface mesh only weakly affect the lowest frequency connectome harmonics. The relationship between the structural connectome and the RSNs is a covered topic (Honey, Ktter, Breakspear, Sporns, 2007, Deco, Jirsa, McIntosh, 2011), and many factors have been shown to influence this relationship, including the topology of the connectome (Goñi et al., 2014; Messé et al., 2015). The important differentiator of the connectome harmonics framework is that it not only uses white matter connectivity (as other studies do) but in addition incorporates local gray matter connectivity of 1-6mm, which we show is critical to obtain a meaningful decomposition of structure into functional patterns on the cortical surface that possibly support RSNs. Notably, our findings that such local gray matter connectivity must account for 30–80% of all cortical connections is consistent with neuroanatomical observations in cats and rodents (Stepanyants et al., 2009, Boucsein, Nawrot, Schnepel, Aertsen, 2011).

###  

 

#### ***Interpretation of local gray matter connectivity constructed from the cortical surface mesh***

Interconnecting cortical surface mesh edges to model gray matter connectivity is relatively common (Spiegler, Jirsa, 2013, Ecker, Ronan, Feng, Daly, Murphy, Ginestet, Brammer, Fletcher, Bullmore, Suckling, Baron-Cohen, Williams, Loth, Consortium, Murphy, 2013, Lo, O’Dea, Crofts, Han, Kaiser, 2015, Spiegler, Abadchi, Mohajerani, Jirsa, 2020, Spiegler, Hansen, Bernard, McIntosh, Jirsa, 2016). The spatial extent of the local connectivity kernel width in our study is consistent with gray matter tracing studies (Gonzlez-Burgos, Barrionuevo, Lewis, 2000, Melchitzky, GonzlezBurgos, Barrionuevo, Lewis, 2001, Braitenberg and Schuz, 2013). Other approaches for constructing local gray matter connectivity from the cortical surface mesh have been proposed. For example, Ecker et al. (2013) used the radius and perimeter of a cortical patch, whose surface is given by a fixed ratio of the total cortical surface, as a proxy for gray matter intrinsic cortico-cortical wiring cost. This cost, they determined, is altered in autism. Other techniques could also be applied, whereby local connectivity between adjacent regions of an atlas-based parcellation is estimated by the boundary length between regions. The analysis of such a framework, through which graph spectral properties and their relationships to connectome harmonics can be observed, is an exciting direction for future studies.

#### ***Role of local gray matter versus long-range white matter connections***

We found that the local gray matter connections encoded in the connectivity of the cortical surface mesh is preponderant for observing high mutual information between low frequency connectome harmonics $\psi_{k\in K=\{7,8,9,10,11\}}$ (as in Atasoy et al., 2016) and the DMN. However, without the presence of long-range interhemispheric connections, spatial maps are restricted to separate hemispheres, and thus whole brain maps are not observed. Yet, our results demonstrate that long-range connections alone are not sufficient for the emergence of whole brain maps when using a high-resolution connectome of several thousands nodes, as they have the side effect of causing brain networks to divide into many isolated components without the formation of large-scale patterns on the cortical surface. This “isolated components” issue occurs because under this condition, the graph is disconnected. Connectedness means mathematically that there exists (at least) a path from one vertex to any other vertex through the graph. Within the high-resolution connectome, the white matter connectivity matrix often does not fulfill this condition; the local connections through the cortical surface mesh alleviates this problem. Hence, our findings emphasize that a careful blend of local and long-range connectivity is necessary for the emergence of functionally meaningful harmonic patterns spanning the whole cortical surface. Our results confirm that connectome harmonics naturally provide a representation, where low frequencies exhibit more robust, stable, and generic patterns across subjects, while high frequency harmonics provide more variable, sensitive, and possibly more subject-specific patterns. Our finding that local connectivity is preponderant for observing high MI between connectome harmonics and the DMN does not invalidate previous studies that retrieved a degree of correlation between the white-matter connectivity and the DMN (Horn et al., 2014), the functional connectivity computed from diffusion models with noise (Honey et al., 2009; Saggio et al., 2016), or more complex non-linear models with time delays (Honey, Sporns, Cammoun, Gigandet, Thiran, Meuli, Hagmann, 2009, Cabral, Hugues, Sporns, Deco, 2011). Rather, it suggests that local gray matter connectivity may also play an important role in the emergence of resting states. Strong reverberating lateral loops present in gray matter microcircuits likely influence the emergence and sustenance of localized cortical activity recorded in EEG and fMRI, and is present in some other work (Honey et al., 2009). These loops are incorporated into the connectome harmonics framework via the local connectivity kernel and our results demonstrate that the incorporation of both local and long-range connections provides an interesting basis to address the role of gray versus white matter in large scale cortical dynamics. Further refinement of the local gray matter connections to include region-specific connectivity profiles shall be examined in the future in order to assess specifically which aspects of gray matter connectivity influence large scale functional networks.

#### ***Relation to disease conditions***

Interestingly, our gradual removal of inter-hemispheric white matter connections can be related to the degeneration occurring in the corpus callosum as observed in cohorts of patients with Huntington’s disease (McColgan et al., 2017). In this condition, lateral callosal degeneration is associated with pre-manifest stages of the disease, and evolves with the disease progression to white matter disruption in medial areas. Strikingly, functional studies show that the DMN remains unaffected by the disease until much later stages when the patient becomes strongly symptomatic (Poudel et al., 2014), an observation which is consistent with our prediction about the effects of callosectomy. Similarly, our disruption of gray matter connectivity, here referred to as anisotropy, could be related to later phases of normal aging, wherein gray matter atrophy is observed (Sala-Llonch et al., 2015), and may in turn induce degenerative disorders (Thompson et al., 2003; Whitwell et al., 2010). In those conditions, gray matter disruptions are often localized to specific brain regions, while in our study they are applied to the whole cortex. Nevertheless, the investigation of these disease specific disruptions through the prism of graph spectral theory is promising (Sihag et al., 2020) and will be the subject of future studies.

#### ***Other uses of graph spectral theory in neurosciences***

Other frameworks utilizing the graph Laplacian on brain structural connectivity rely on coarser resolution connectomes (Raj, Kuceyeski, Weiner, 2012, Huang, Bolton, Medaglia, Bassett, Ribeiro, Ville, 2018, Preti and Van De Ville, 2019, Tewarie, Abeysuriya, Byrne, O’Neill, Sotiropoulos, Brookes, Coombes, 2019), defined by atlases of larger brain regions, each of which usually spanning several cubic centimeters (Desikan et al., 2006; Destrieux et al., 2010; Glasser et al., 2016). Employing such atlases alleviates the problem of ”isolated components” because isolated subnetworks do not emerge given the chosen size of brain areas. An important limitation of the parcellated approach is that it yields sharp boundaries between coarse brain regions, such that two neighbouring points on the cortex can be affiliated with two different brain areas, and resulting in whole brain connectivity matrices reflecting only the white matter tracks and not adequately taking into account gray matter conduction. The effect of local gray matter versus long-range white matter connectivity on large-scale brain dynamics using coarse connectomes have nonetheless been investigated using neural field models (Proix et al., 2016). It has been suggested that white matter connectivity shapes the cortical slow rhythms while faster dynamics are dependent on short-range connections. Hence, further extension of these studies to the use of high-resolution connectomes such as utilized here and in (Atasoy, Donnelly, Pearson, 2016, Atasoy, Deco, Kringelbach, Pearson, 2017 can be an important direction for future research.

#### ***Different methods for connectome reconstruction***

Different approaches can also be utilized in reconstructing a weighted connectome from tractography streamlines (Smith, Tournier, Calamante, Connelly, 2015, Donahue, Sotiropoulos, Jbabdi, Hernandez-Fernandez, Behrens, Dyrby, Coalson, Kennedy, Knoblauch, Essen, Glasser, 2016, Horn, Blankenburg, 2016). For example, in (Horn and Blankenburg, 2016) each fiber weight decays exponentially with distance of track bounds to cortical surface. In this study, we have only excluded fiber tracks whose endpoints are farther than 5mm from the cortical surface. We acknowledge that streamline end-points localization are associated with some degree of spatial error, due to the limitations of tractography and accumulation of errors during streamline propagation (Jones et al., 2013). Some degree of spatial smoothing on the high-resolution connectome could be performed to alleviate this spatial error, and will be incorporated to future releases of the framework. It must also be noted that high-resolution connectomes are computationally expensive to create and manipulate. Specifically, since the eigendecomposition of a matrix with *n* entries has a computational complexity of $O(n^{3})$, it becomes quickly intractable for matrix sizes above 30,000 vertices. For reference, the algorithm took 1 to 5 minutes to converge using the 20,484-by-20,484 matrix corresponding to *fsaverage5* on a laboratory workstation.

In line with previous studies (Besson et al., 2014; Donahue et al., 2016), we found the intersection between white matter and gray matter cortical surface to yield the most effective connectivity matrix, since the intersection between the white-matter fiber tracks and this cortical mesh are more reliable and anatomically more meaningful than their intersection with the pial surface. The use of template datasets further introduces possible errors in the intersection between the cortical surface and the tracks. These templates allowed our findings to generalize well at the group level, but with the drawback of losing inter-individual specificity, which may be crucial for medical applications. This further highlights an important difference with the original work from Atasoy et al. (2016) who used subject specific tractography streamlines with their cortical surface meshes in the same native space (n=10 subjects). Instead, we used normalized tractography streamlines from the 169 subjects of the Gibbs dataset (Horn and Blankenburg, 2016) with template cortical meshes provided in FreeSurfer. Tracks and surface were registered in MNI coordinates and visually checked for proper alignment with the 20.7 million streamlines from the Gibbs dataset. The track-mesh intersection algorithm presented here results in a connectome with more than 10 million entries, using a minimal track length constraint of 10 mm. On the one hand, this approach has the advantage of a much larger cohort and higher quality tractography streamlines that have been validated across several white matter atlases (Horn and Blankenburg, 2016). On the other hand, our use of template cortical surface meshes, whose geometry in template space may not perfectly correspond to the normalized streamline bounds in native space, represents a sensible pitfall. For future studies, it will be important to favor the use of cortical surfaces in native space so that streamline bounds are perfectly aligned to the cortical mesh as we did for the HCP subject in Supplementary Materials.

#### ***Relation to Spherical Harmonics and Surface Laplacian***

Connectome harmonics can also be viewed as an extension of spherical harmonics, which correspond to the eigenfunctions of the Laplace operator applied to a sphere. In the absence of any long-range white matter connectivity, connectome harmonics of each hemisphere, deriving only from the gray matter connectivity, correspond to spherical harmonics mapped to the cortical surface. Similar decompositions of cortical activity patterns into spherical harmonics have been previously brought by (Jirsa et al., 2002; Robinson et al., 2016), whereby each brain hemisphere is modeled as a perfect sphere. A similar approach based on spherical harmonics has also been utilized in electroencephalogram (EEG) and magnetoencephalography (MEG) source reconstruction, where the scalp is modelled as the homogeneous surface of a sphere on which the electrical signals propagate isotropically from a neuronal source (Nunez, Srinivasan, 2006, Petrov, 2012, Carvalhaes, de Barros, 2015). While conceptually similar, the connectome harmonics framework extends other spherical harmonic approaches and embeds the full structural connectivity of the human brain by taking into account both local gray matter and long-range white matter connectivities.

Lastly, we point out that as connectome harmonics provide an extension of the Fourier basis and spherical harmonics to the human connectome, they also yield a frequency-specific functional basis which enables the represention of any pattern of cortical activity. This important property of connectome harmonics has been utilized for the spatial frequency analysis of functional magnetic resonance imaging (fMRI) data, which has demonstrated crucial differences in the signatures of different brain states (Atasoy, Deco, Kringelbach, Pearson, 2017, Atasoy, Roseman, Kaelen, Kringelbach, Deco, Carhart-Harris, 2017, Atasoy, Vohryzek, Deco, Carhart-Harris, Kringelbach, 2018).

## 5 Data and code availability

The data used in this study is publicly available. Tractography streamlines are part of the open source Gibbs connectome dataset (https://www.nitrc.org/projects/gibbsconnectome/) and cortical surface meshes are provided by FreeSurfer software suite (https://surfer.nmr.mgh.harvard.edu/). The framework for constructing connectome harmonics is integrated to the open source SCRIPTS pipeline, in the branch *ConnectomeHarmonics* (https://github.com/ins-amu/scripts/).

Template connectome harmonics $\psi_{k\in K=1\ldots 100}$ are provided in .mat format alongside intermediate graph measures for the cortical surface meshes *cvs_avg35_inMNI152, fsaverage4* and *fsaverage5*, using the Gibbs connectome with default framework parameters from Table 1. The files are hosted on Zenodo at https://zenodo.org/record/4027989.

## Conflict of interest

The authors declare the absence of conflict of interest. Funding sources had no role in study design, data analysis, decision to publish, or preparation of the manuscript.

## CRediT authorship contribution statement

**Sébastien Naze:** Conceptualization, Methodology, Software, Validation, Formal analysis, Writing - original draft, Writing - review & editing, Visualization, Project administration. **Timothée Proix:** Conceptualization, Methodology, Software, Validation, Writing - original draft, Writing - review & editing. **Selen Atasoy:** Conceptualization, Methodology, Validation, Writing - review & editing. **James R. Kozloski:** Conceptualization, Resources, Writing - review & editing, Funding acquisition.

## Appendix A Supplementary materials

Supplementary Data S1 Supplementary Raw Research Data. This is open data under the CC BY license http://creativecommons.org/licenses/by/4.0/ Supplementary Data S1
