# Supplementary material for: Robustness of connectome harmonics to local gray matter and long-range white matter connectivity changes
Source: Neuroimage. 2021 Jan 1;224:117364. doi: 10.1016/j.neuroimage.2020.117364 (PMC7779370; doi:10.1016/j.neuroimage.2020.117364)
Supplement: Supplementary Data S1 — Supplementary Raw Research Data. This is open data under the CC BY license http://creativecommons.org/licenses/by/4.0/ [file mmc1.pdf]

Supplementary Materials (Naze et al. 2020)

This document present supplementary materials for the article:

Naze S., Proix T., Atasoy S., Kozloksi J. R. (2020) Robustness of connectome harmonics to local gray matter and long-range white matter connectivity changes. *NeuroImage*. DOI: 10.1016/j.neuroimage.2020.117364.

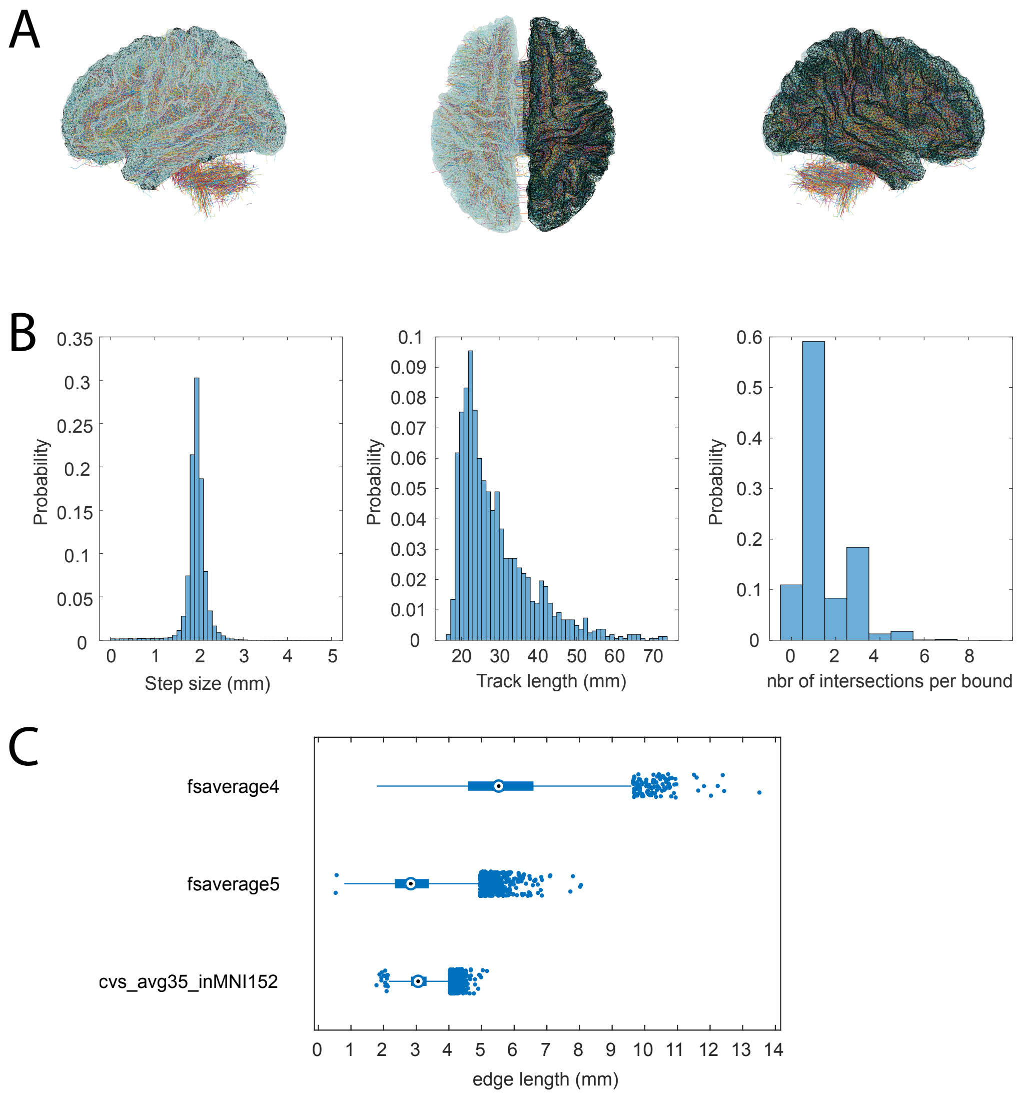

Supplementary Figure 1: Tracks and cortical surface mesh statistics

A) Alignment of template cortical surface mesh from Freesurfer to tractography streamlines from the Gibbs connectome. B) Tracks step size (left), track length (middle), and number of intersection between track bound and cortical surface mesh for 10000 randomly selected tracks of the Gibbs dataset. C) Cortical surface mesh edge length distribution for the 3 template meshes shown as box plots. Circled dot shows the mean, boxes span from the 25th to the 75th percentiles and whiskers extend to all values not considered outliers. Outliers are shown by dots away from whiskers.

Supplementary Text 1

Diffusion MRI acquisition summary from Horn and Blankenburg (2016) [1]

High angular diffusion images (HARDI) were used for global tracking that had been acquired with a diffusion-sensitive multiband sequence (TR = 2400 ms, TE = 85 ms, voxel-size of 2 x 2 x 2 mm^3, 64 slices). An effective mean b-value of 1500 s/mm^2 was used for 137 diffusion encoding directions. In addition, 9 volumes with very low diffusion weighting (b-value = 5 s/mm^2) equally distributed throughout the scan were acquired (b0-images). Acquisition time of DTI images lasted 5.58 min. Details of the MR protocol are available online (`http://fcon_1000.projects.nitrc.org/indi/enhanced/mri_protocol.html`). For anatomical segmentation and estimation of a group template, high-resolution anatomical images were used that had been acquired using a standard magnetization-prepared rapid gradient-echo (MPRAGE) sequence (TR = 1900 ms, TE = 2.52 ms, isotropic voxel-size of 1 x 1 x 1 mm^3, 176 slices with a distance factor of 50% resulting in a slice gap of 0.5 mm).

Tractography summary from Horn and Blakenburg (2016) [1]

To estimate structural connectivity, the Gibbs' tracking-approach [2] as implemented in the DTI and Fiber Tools for SPM [3] was used to reconstruct the global fiber-dataset directly from the DTI volumes. In this approach, based on the raw HARDI data, a piecewise approximation of neuronal pathways is achieved by annealing small cylinders to chains that finally form fiber tracts. These cylinders are simulated as particles moving in a Brownian motion fashion, and their polymerization to chains is attained by slowly reducing a simulated temperature. In a Bayesian framework, a signal $\hat{S}^*$ is simulated by a spatially distributed sum of all cylinders and optimized iteratively during cylinder annealing to best explain the empirical MR-signal $\hat{S}$. The following parameters were chosen: Starting temperature: 0.1, stop temperature: 0.001, numbers of steps: 50, numbers of iterations: 5 x 108, particle width: 1 mm, particle length: 1 mm, weight (unitless): 0.058, and density penalty: 0.2. Global tracking was performed on a tracking mask that was based on the white matter volume of the anatomical volumes (which was co-registered to the b0-images). These parameters represent the default dense parameter set [3] and were kept identical for fiber tracking in all subjects. On average 1.23 x 105 fibers were obtained for each subject.

References

[1] A. Horn and F. Blankenburg. Toward a standardized structuralfunctional group connectome in MNI space. *NeuroImage*, 124:310–322, Jan. 2016.

[2] B. W. Kreher, I. Mader, and V. G. Kiselev. Gibbs tracking: a novel approach for the reconstruction of neuronal pathways. *Magnetic Resonance in Medicine: An Official Journal of the International Society for Magnetic Resonance in Medicine*, 60(4):953–963, 2008. Publisher: Wiley Online Library.

[3] M. Reisert, I. Mader, C. Anastasopoulos, M. Weigel, S. Schnell, and V. Kiselev. Global fiber reconstruction becomes practical. *NeuroImage*, 54(2):955–962, Jan. 2011.

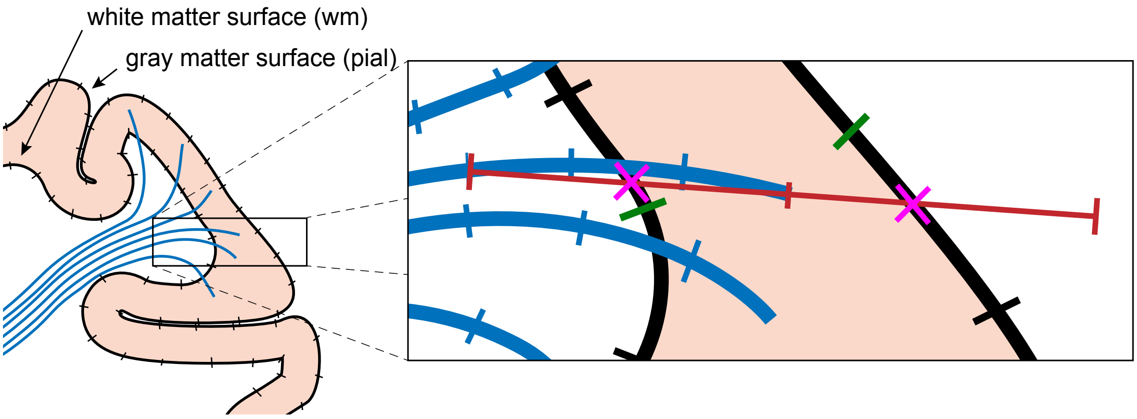

Supplementary Figure 2: Illustration of track-mesh intersection routine to compute *high-resolution* connectomes. Schematic of the white matter and gray matter cortical meshes (black), and six tracks (blue) from the Gibbs connectome. Inset: magnified area illustrating track mesh intersection routine (see **Methods**). Red segment: linear extension of track. Pink crosses: coordinates of intersections with meshes. Green symbols: closest vertex to the intersection with the white matter mesh .

Table 1: Mapping of Resting State Networks onto the Desikan-Killiany atlas.

Resting State Network	Region name code
Default Mode Network (DMN)	inferiorparietal, isthmuscingulate, middletemporal, parahippocampal, parsorbitalis, posteriorcingulate, precuneus, rostralanteriorcingulate, superiorfrontal, superiortemporal
Sensory-Motor (SM)	precentral, postcentral, paracentral, transversetemporal, insular
Fronto-Parietal (FP)	caudalanteriorcingulate, fusiform, inferiortemporal, rostralmiddlefrontal, parstriangularis
Visual (Vis)	lateraloccipital, pericalcarine, cuneus, lingual
Limbic (L)	entorhinal, lateralorbitofrontal, medialorbitofrontal, frontalpole, temporalpole
Ventral Attention (VA)	bankssts, parsopercularis, supramarginal
Dorsal Attention (DA)	caudalmiddlefrontal, superiorparietal

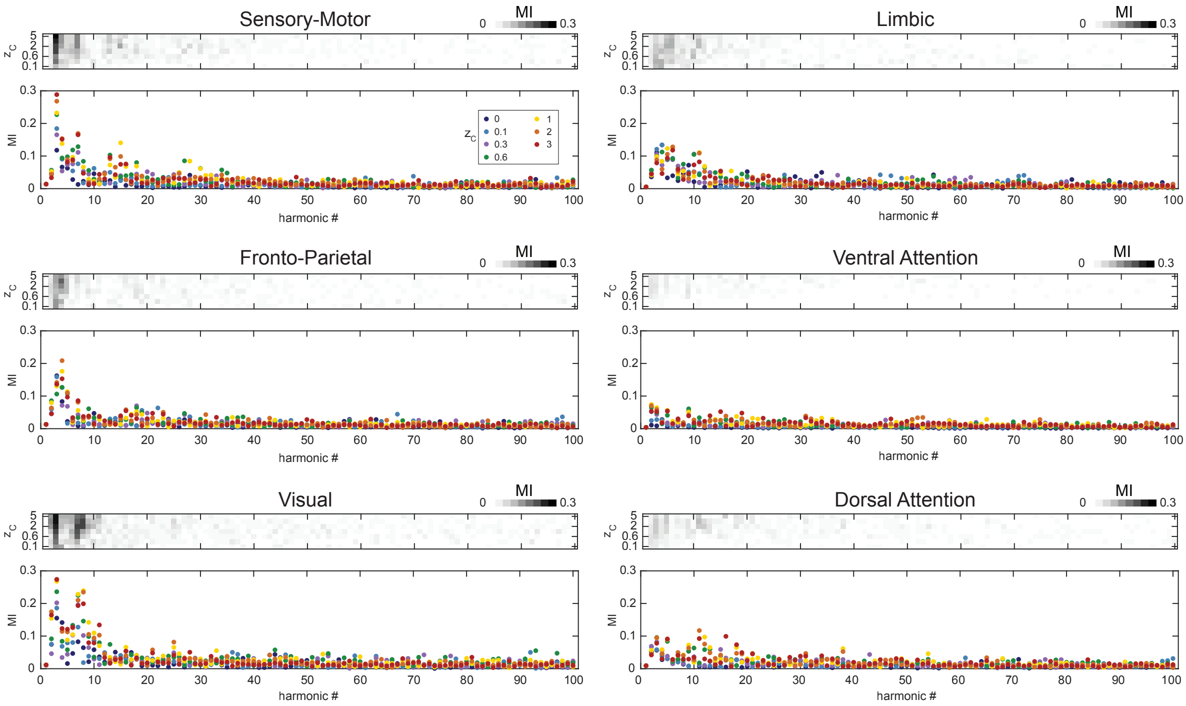

Supplementary Figure 3: Mutual Information between connectome harmonics $\psi_{k \in K=\{1,\ldots,100\}}$ and other resting state networks.

Mutual information (MI) is computed between the first 100 harmonics and several resting state networks (RSN) for different values of matrix weight threshold z_C as in main text Figure 2. RSN are mapped on the Desikian-Killiany atlas: Sensory-motor network: pre-central, post-central, paracentral and transverse temporal regions; Fronto-parietal network: caudal anterior cingulate, rostral middle frontal, inferior temporal, fusiform regions; Visual: lateral occipital, cuneus, pericalcarine and lingual regions; Limbic: entorhinal, lateral orbito-frontal, medial orbito-frontal; Ventral attention: pars opercularis, insular regions; Dorsal attention: superior parietal, caudal middle frontal regions.

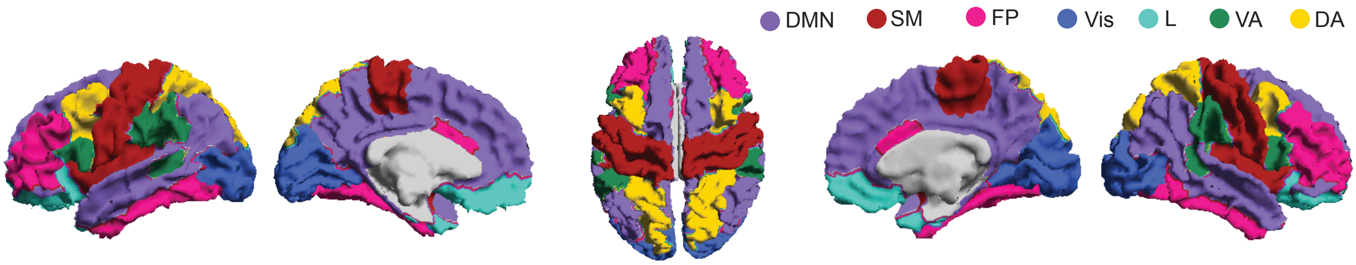

Supplementary Figure 4: Masks of resting state networks (RSN) mapped onto the Desikan-Killiany atlas.

DMN: Default Mode; SM: Sensory-Motor; FP: Fronto-Parietal; Vis: Visual; L: Limbic; VA: Ventral Attention; DA: Dorsal Attention.

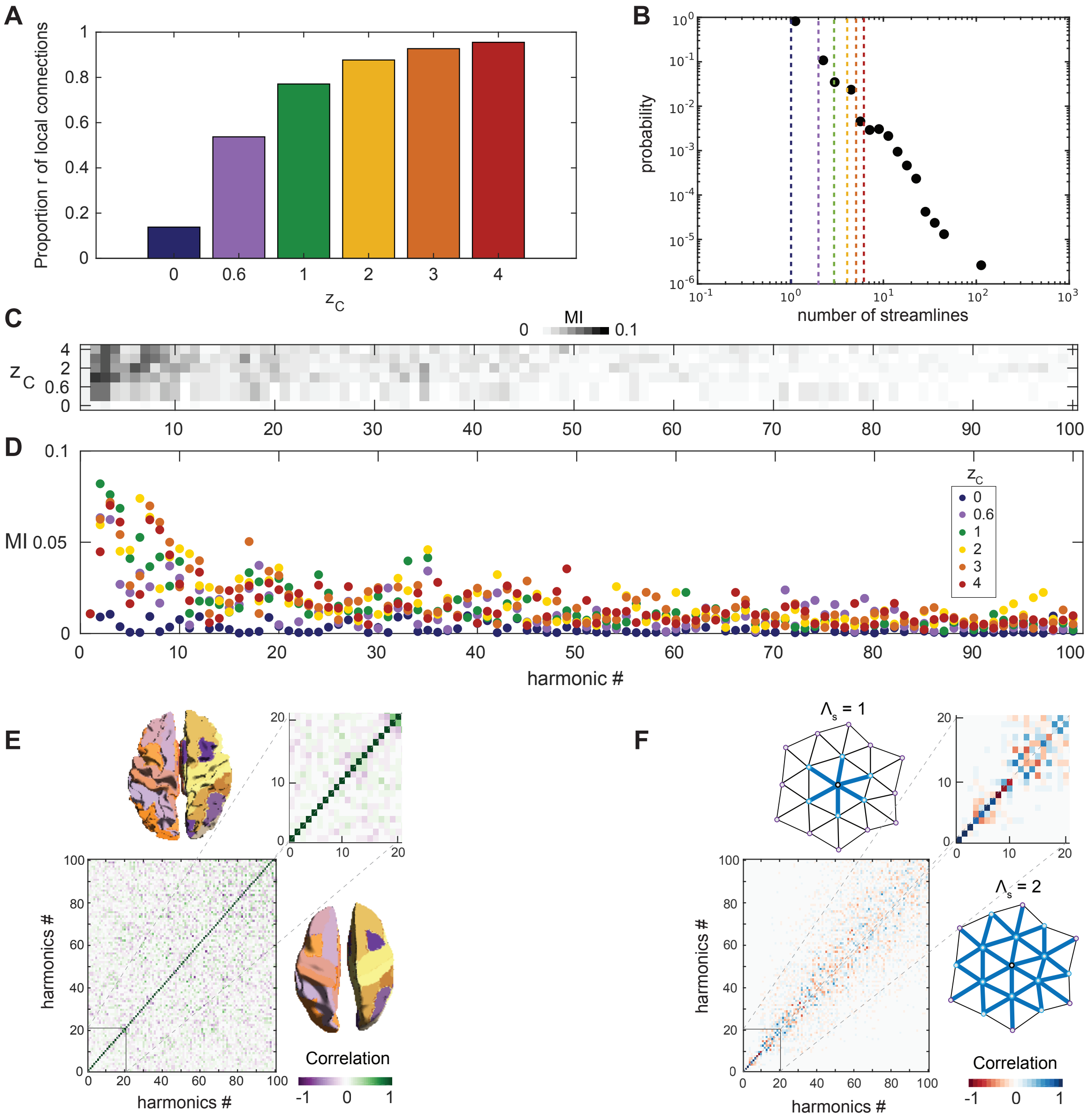

Supplementary Figure 5: Reproduction of main findings in HCP subject 100307.

(A) Ratio r of local gray matter connections in the adjacency matrix A of the graph Laplacian, for different threshold value z_C applied to the long-range white matter connectivity.

(B) Distribution of connectome weights (number of white matter streamlines between vertices) in log-log scale, described as probability given that the weight is positive ($P(W|W > 0)$). Vertical lines correspond to z_C values with same color code as A).

(C-D) Mutual information (MI) between the first 100 connectome harmonics and the DMN for a range of z_C resulting in a range of proportions of local to long-range connections. Color code is consistent across panels.

(E) Pearson correlation between 100 first connectome harmonics in coarse-resolution atlas space, of the unsmoothed ($f_i = 0$) vs. smoothed ($f_i = 21$) WMGM cortical surface meshes.

(F) Pearson correlation between 100 first connectome harmonics in high-resolution vertex space, of local connectivity kernel widths $\Lambda_s = 1$ vs. $\Lambda_s = 2$ nearest neighbors. In both **(E)** and **(F)**, z_C is set to 1.

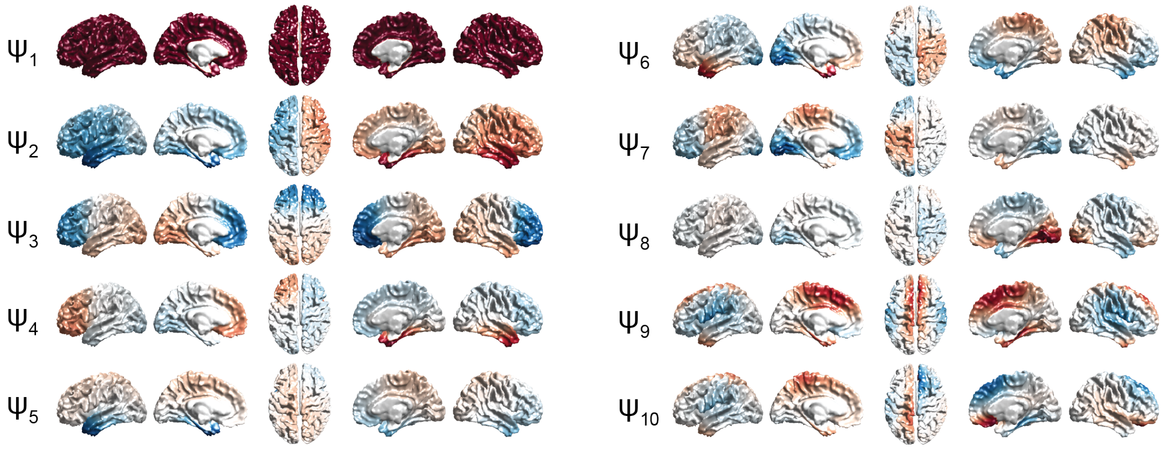

Supplementary Figure 6: Projection of connectome harmonics $\psi_{k\in K=\{1,\ldots,10\}}$ onto cortical surface mesh

Original: First 10 connectome harmonics $\psi_{k\in K=\{1,\ldots,10\}}$ based on unaltered local and long-range connectivity, with white matter weight threshold $z_C = 1$ and other default parameters presented in main text Table 1.

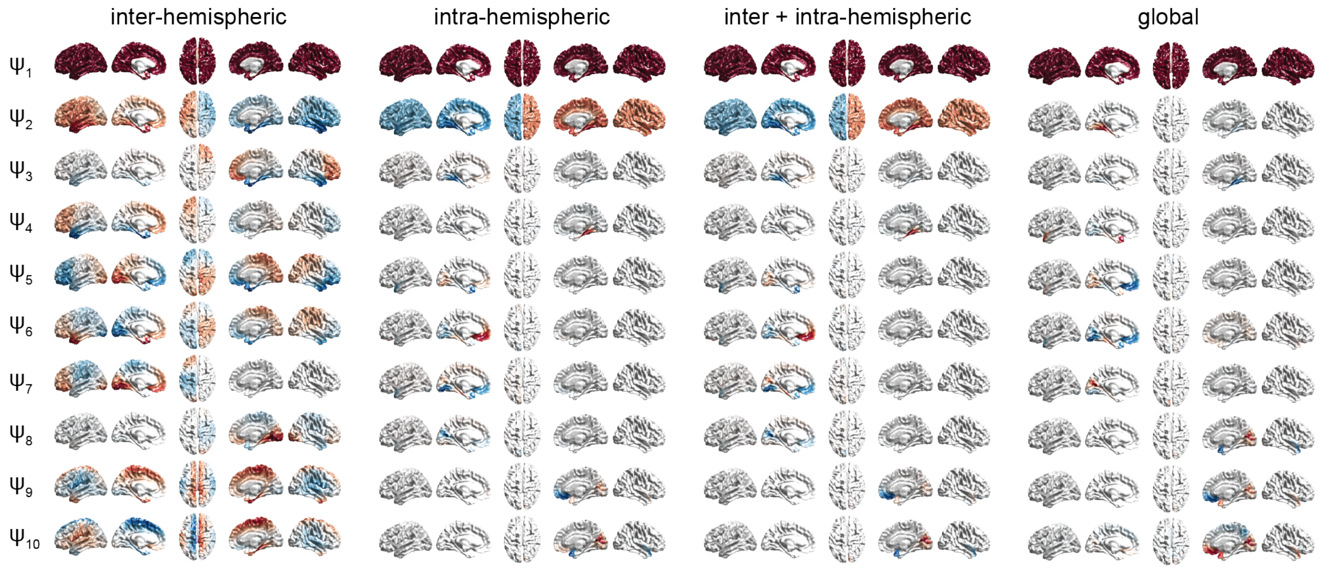

Supplementary Figure 7: Projection of connectome harmonics $\psi_{k\in K=\{1,\ldots,10\}}$ onto cortical surface mesh for different types of randomizations

Randomization is performed on subgraphs corresponding to inter-hemispheric connections, intra-hemispheric connections, inter-hemispheric and intra-hemispheric connections randomized separately, and then assembled, or randomized together (global). Framework parameters set to default from main text Table 1. *nb: Eigenmodes are fundamentally identical under sign reversal, i.e. spatial pattern is relevant over color code.*

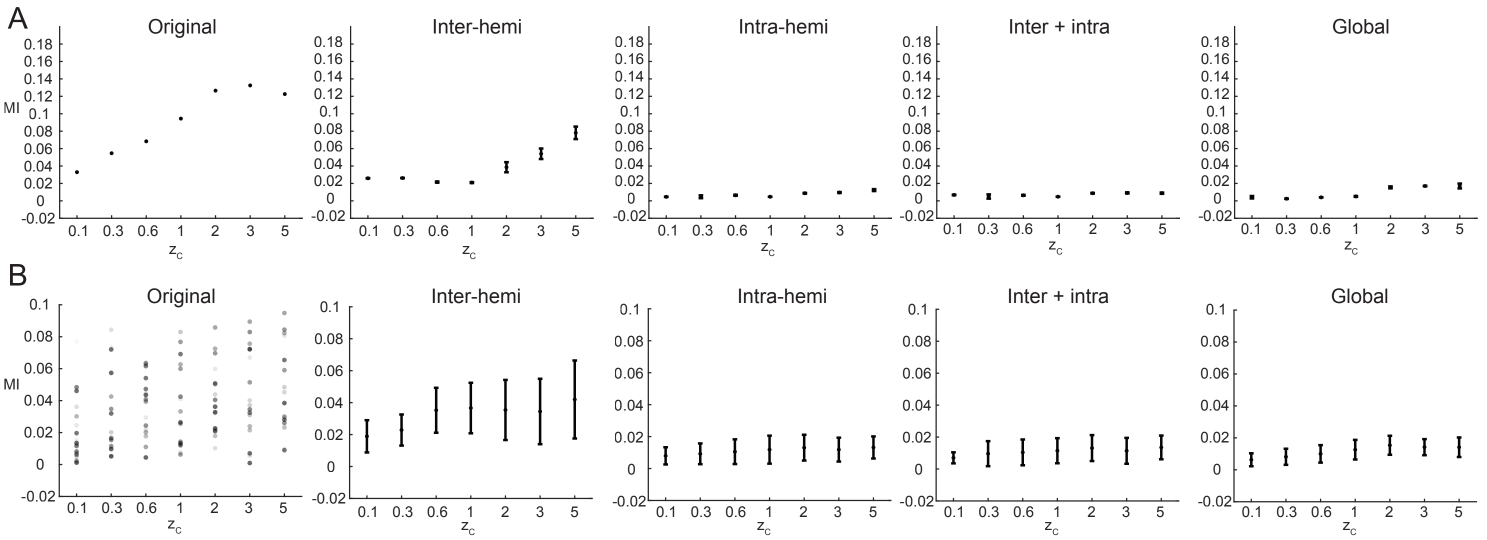

Supplementary Figure 8: Mutual information with DMN of single and ranges of harmonics are affected by randomizations of long-range connectomes.

A) Mutual information (MI) between the DMN and the 3rd harmonic for the different randomization schemes and values of z_C. B) MI between the DMN and a larger set of harmonics ($\psi_{k \in K=\{2,\dots,20\}}$) for the different randomization schemes and values of z_C. When no randomization is performed (Original), each dot is the MI of an harmonic and the DMN. When randomization is performed, MI are shown as mean (dot) and standard deviation (whiskers) over 100 samples.

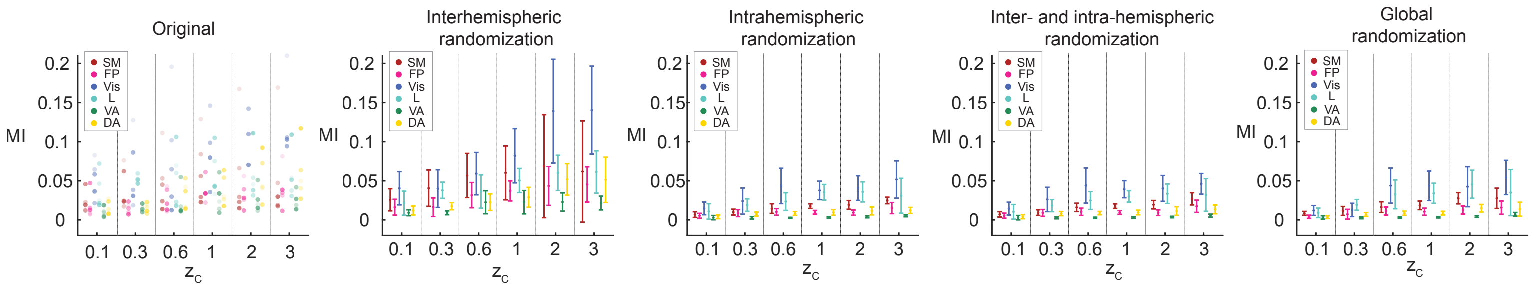

Supplementary Figure 9: Mutual Information (MI) between connectome harmonics and the other resting state networks (RSN) for several randomized versions of long-range connectivity.

Mean and standard deviation of connectome harmonics' $\psi_{k \in K=\{7,8,9,10,11\}}$ MIs for different proportion of local:long-range connections (parameterized by the adjacency weight threshold z_C), and randomizations of the long-range connectivity as in main text **Figure 3**. Other framework parameters set to default from maon text Table 1. SM: Sensory-Motor; FP: Fronto-Parietal; Vis: Visual; L: Limbic; VA: Ventral Attention; DA: Dorsal Attention.

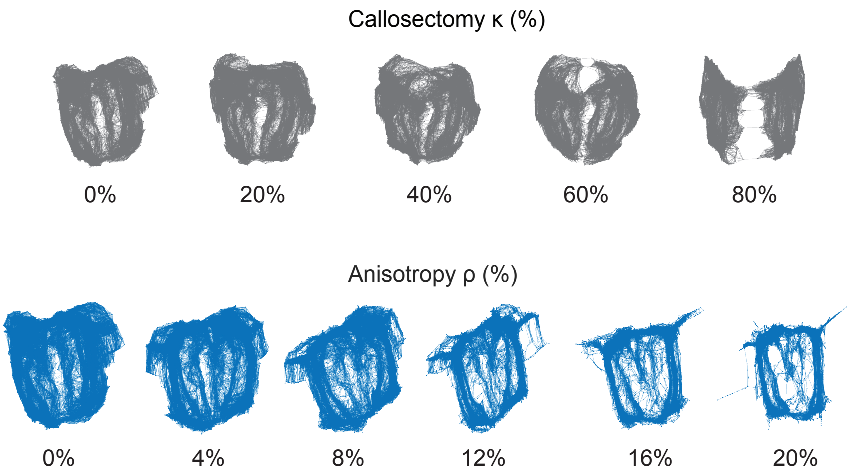

Supplementary Figure 10: Illustrations of graph structures for altered combined connectomes. Graph representations of combined (i.e. local and long-range connectivity for gradual removal of inter-hemispheric connections (top, referred as *callosectomy*) and cortical surface mesh edges (bottom, referred as *anisotropy*)

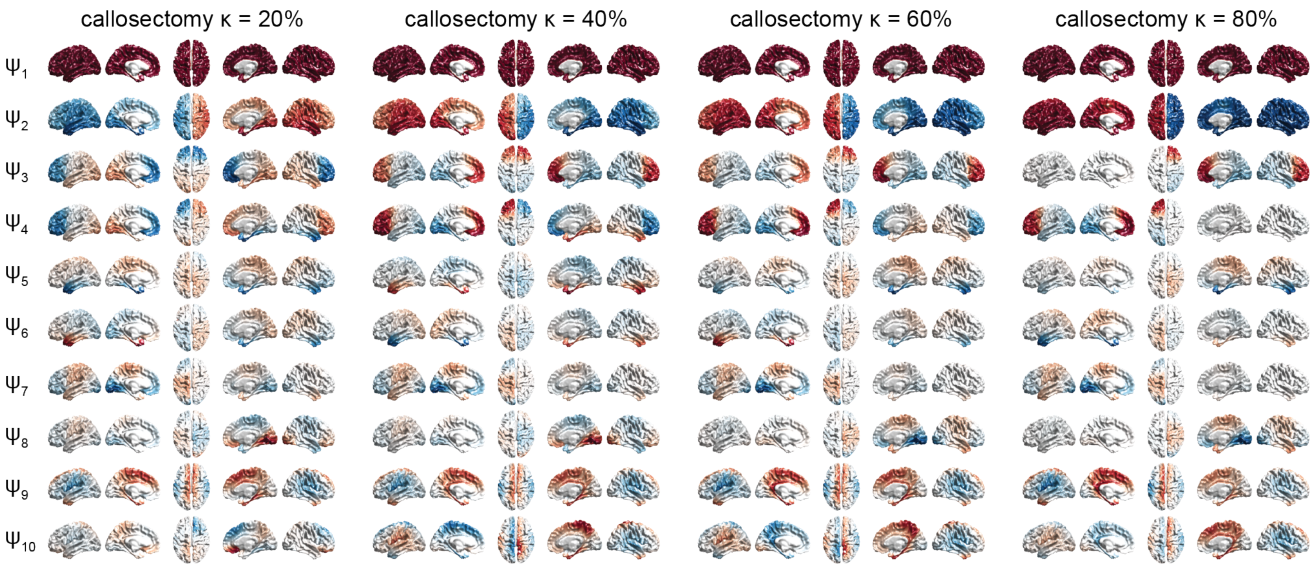

Supplementary Figure 11: Projection of connectome harmonics $\psi_{k \in K=\{1,\ldots,10\}}$ onto cortical surface mesh for gradual fractions of inter-hemispheric connections trimming.
Callosectomy: proportion κ of long-range inter-hemispheric white matter connections trimmed by descending order of track lengths. Other framework parameters set to default from main text Table 1. *nb: Eigenmodes are fundamentally identical under sign reversal, i.e. spatial pattern is relevant over color code.*

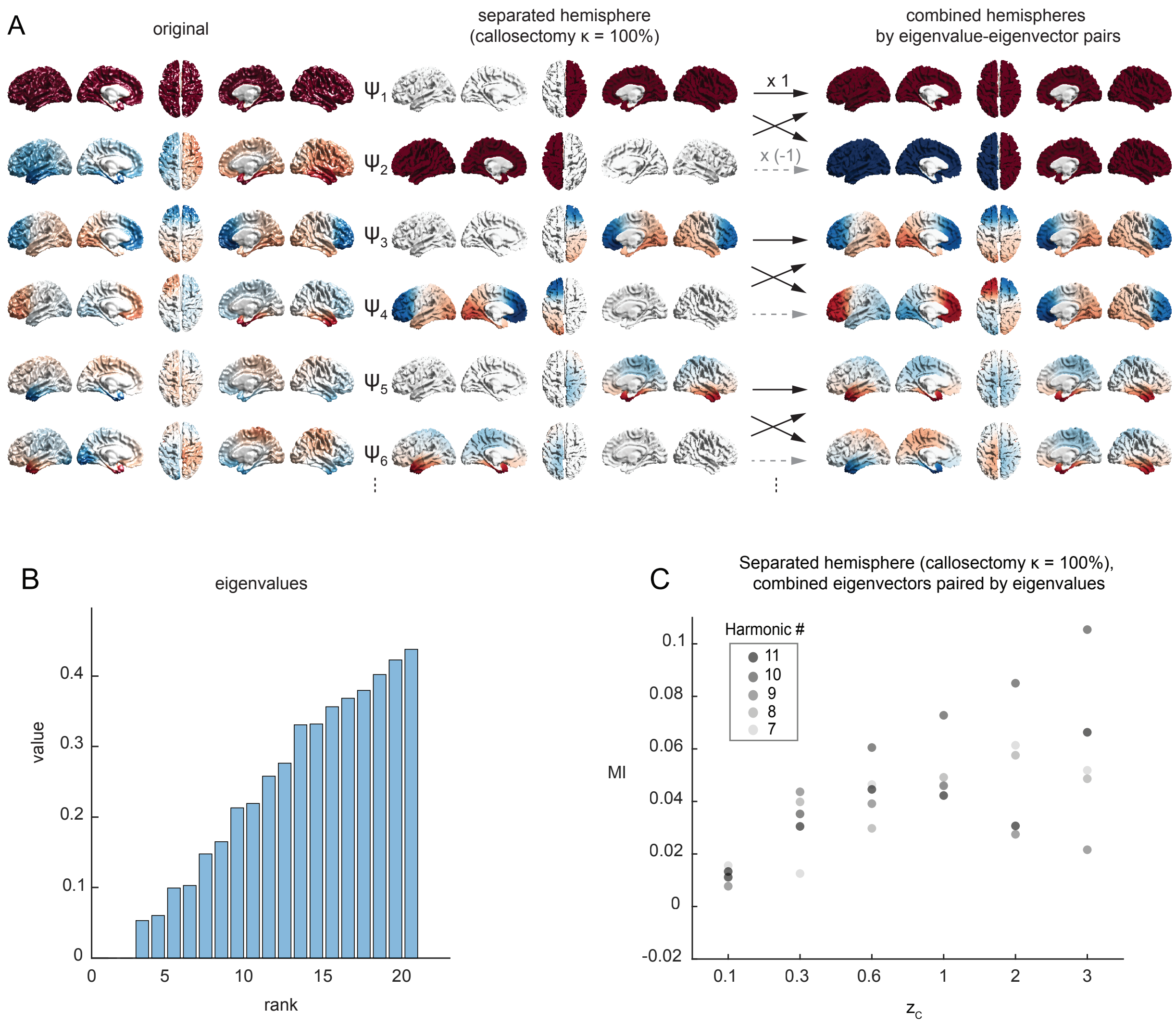

Supplementary Figure 12: Reconstruction of harmonics from separated hemispheres.

A) Illustration of the reconstruction of harmonics from separated hemispheres. Harmonics are assembled by pairs of eigenvectors, computed on each hemisphere separately and assembled by corresponding eigenvalues. Since the sign of the eigenvector is attributed randomly, harmonics are assembled using same $(\times 1)$ and reverse $(\times(-1))$ signs, therefore for each pair of eigenvectors, two whole brain harmonics are generated. B) 20 first eigenvalues forming the spectrum of the combined connectome in the case of separated hemispheres. Note how eigenvalues a grouped 2-by-2 , one for each hemisphere in each pair. C) MI between DMN and harmonics $\psi_{k\in K=\{7,8,9,10,11\}}$ for different values of z_C, in the case of 100% callosectomy $(\kappa = 1)$ whereby harmonics are computed on each hemisphere separately and combined by eigenvalue-eigenvector pairs.

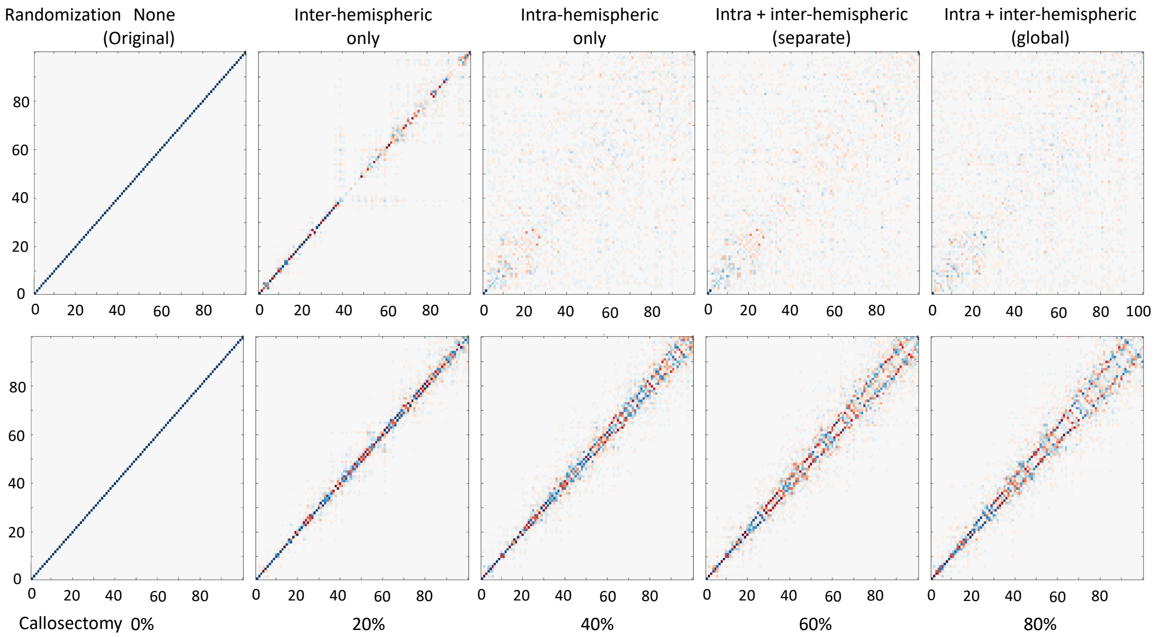

Supplementary Figure 13: Correlations between connectome harmonics $\psi_{k \in K=\{1,\ldots,10\}}$ for connectome randomizations and callosal trimming κ.

top: Correlation matrices of connectome harmonics $\psi_{k \in K=\{1,\ldots,100\}}$ between Original *(None)* and different randomization types. *bottom:* Correlation matrices of connectome harmonics $\psi_{k \in K=\{1,\ldots,100\}}$ between Original *(0%)* and increasing degrees of callosal trimming κ (i.e. callosectomy).

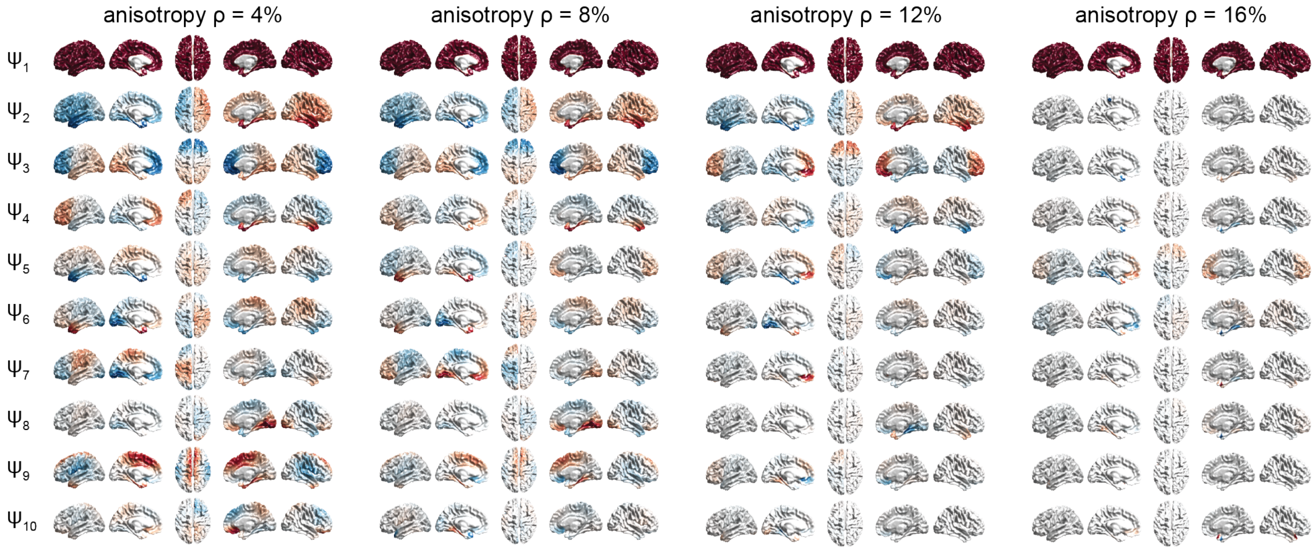

Supplementary Figure 14: Projection of connectome harmonics $\psi_{k \in K=\{1,\dots,10\}}$ onto cortical surface mesh for gradual fraction of local trimming ρ.

Anisotropy: proportion ρ of local mesh connections are trimmed randomly. Other framework parameters are set to default from Table 1. *nb: Eigenmodes are fundamentally identical under sign reversal, i.e. spatial pattern is relevant over color code.*
